# Hippocampal Synaptic Expansion Induced by Spatial Experience in Rats Correlates with Improved Information Processing in the Hippocampus

**DOI:** 10.1371/journal.pone.0132676

**Published:** 2015-08-05

**Authors:** Mariana Carasatorre, Adrian Ochoa-Alvarez, Giovanna Velázquez-Campos, Carlos Lozano-Flores, Sofía Y. Díaz-Cintra, Víctor Ramírez-Amaya

**Affiliations:** 1 Department of “Neurobiología del Desarrollo y Neurofisiología, Instituto de Neurobiología”, Universidad Nacional Autónoma de México, Querétaro, México; 2 Department of “Neurobiología Conductual y Cognitiva, Instituto de Neurobiología, Universidad Nacional Autónoma de México”, Querétaro, México; 3 Departament of “Microbiología, Maestría en Neurometabolismo & Maestría en Nutrición Humana, Facultad de Ciencias Naturales, Universidad Autónoma de Querétaro, Querétaro, México; Centre national de la recherche scientifique, University of Bordeaux, FRANCE

## Abstract

Spatial water maze (WM) overtraining induces hippocampal mossy fiber (MF) expansion, and it has been suggested that spatial pattern separation depends on the MF pathway. We hypothesized that WM experience inducing MF expansion in rats would improve spatial pattern separation in the hippocampal network. We first tested this by using the the delayed non-matching to place task (DNMP), in animals that had been previously trained on the water maze (WM) and found that these animals, as well as animals treated as swim controls (SC), performed better than home cage control animals the DNMP task. The “catFISH” imaging method provided neurophysiological evidence that hippocampal pattern separation improved in animals treated as SC, and this improvement was even clearer in animals that experienced the WM training. Moreover, these behavioral treatments also enhance network reliability and improve partial pattern separation in CA1 and pattern completion in CA3. By measuring the area occupied by synaptophysin staining in both the *stratum oriens* and the *stratun lucidum* of the distal CA3, we found evidence of structural synaptic plasticity that likely includes MF expansion. Finally, the measures of hippocampal network coding obtained with catFISH correlate significantly with the increased density of synaptophysin staining, strongly suggesting that structural synaptic plasticity in the hippocampus induced by the WM and SC experience is related to the improvement of spatial information processing in the hippocampus.

## Introduction

Behavioral experience promotes structural synaptic plasticity [1]. One remarkable example is the observation that overtraining rats in the Morris water maze (WM) spatial task induces mossy fiber (MF) expansion in the hippocampal CA3 region [2, 3, 4]. This is observed across different rat strains with slightly different remodeling dynamics [4] and in mice [5] in response to environmental enrichment and fear conditioning [6, 7].

It has been proposed that these structural synaptic changes underlie long-term spatial memory formation [8, 9] of the acquired information [4]. However, even when the MF projection is endowed with synaptic plasticity, its sparse synapse density in CA3 pyramidal neurons [10] and the sparse activity of the dentate gyrus (DG, [11]) make it unlikely that the MF-CA3 synapses store information in long-term memory [12]. Conversely, it is possible that the function of the MFs is to direct the encoding of new information in the CA3-CA3 recurrent network, which operates as an auto-associative memory system capable of storing information [13, 14, 15]. For encoding, the MFs promote pattern separation in the CA3 network [13, 16]; this process transforms similar inputs into less-overlapping outputs, allowing us to discriminate between similar experiences and store them separately [17, 18]. Then later, during retrieval, even when a partial or distorted input is presented to the animal, the CA3 network is able to perform pattern completion [17, 18], but this process is thought to be directed by the perforant pathway [13].

Both pattern separation and pattern completion are information-processing functions of the hippocampal network, and they are fundamental features of episodic memory. These functions are also of great relevance for efficient information processing in the brain [18]; thus, any imbalance in these processes may underlie disease states [19] as well as neurocognitive aging [20]. The goal of the present work was to determine whether or not the behaviorally induced structural synaptic plasticity in the hippocampus affects spatial pattern separation.

**Fig 1:**
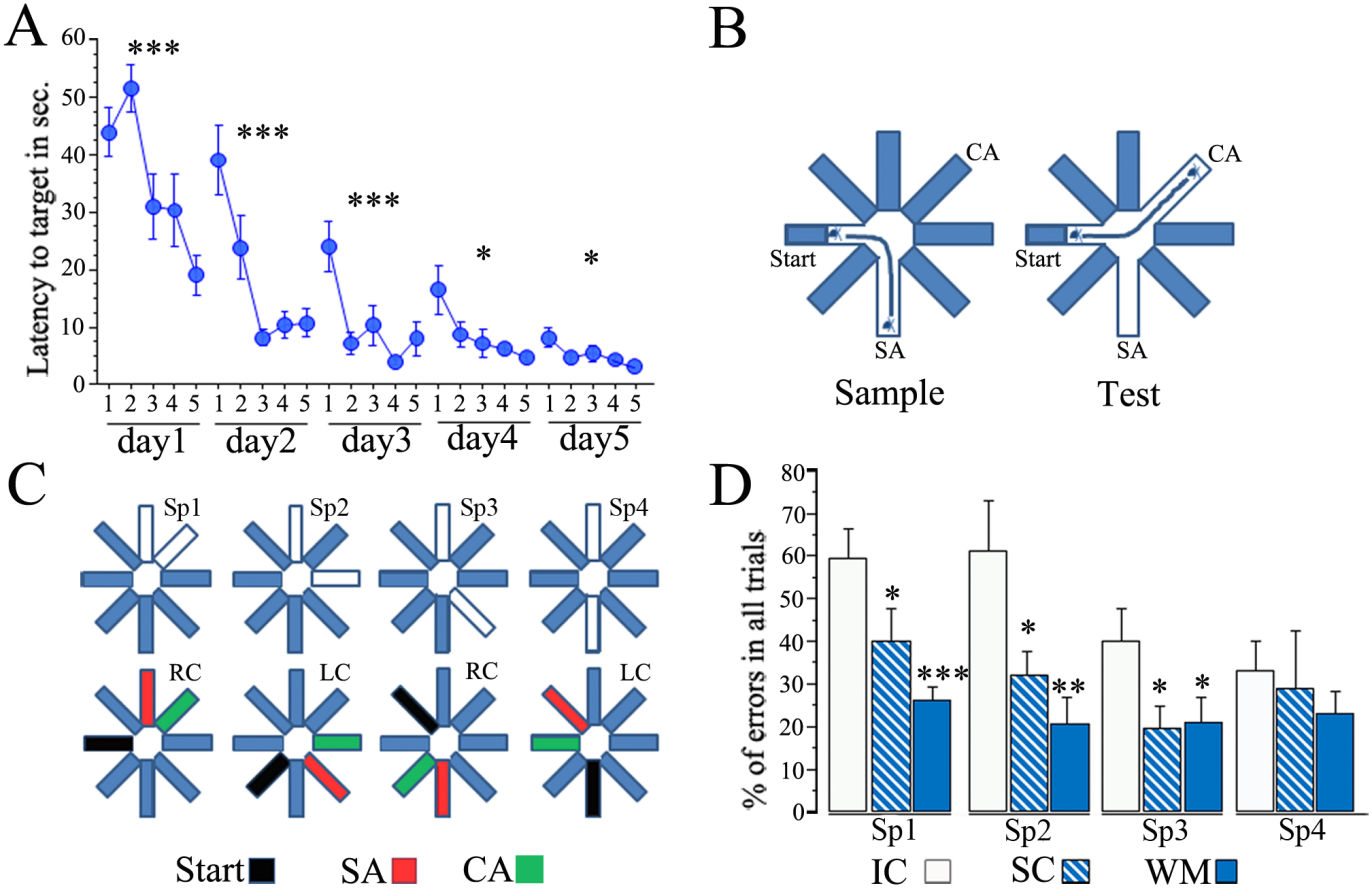
Spatial pattern separation in the DNMP task improves after water maze and swimming treatment. (A) Performance of animals during training in the Morris Water Maze task is expressed as the latency to reach the target platform; each point represents the average latency to reach the target from each pair of trials (5 pairs) from a total of 10 trials each animal underwent each daily session. The animals were trained during 5 sessions that occurred during 5 consecutive days. The trained animals showed a significant decrease in their latency to reach the target between trial pairs each day, particularly during the first 3 days (*** p<0.001, *p<0.05, repeated measures ANOVA). (B) The DNMP task consists of placing the animal in the middle of the start arm and then allowing it to find food at the end of the sample arm (SA), this occurs during the sample phase; during the next phase the animal is release in the same place but it now needs to find the choice arm (CA), which is the only place where food is now available and is open only in this choice phase. To solve the task the animal needs to distinguish the location of the newly available corridor from that one visited in the previous trial and the difficulty comes with the proximity of the 2 arms. (C) Four different separations between the SA and CA were tested (upper diagram); in Sp1 the SA and CA were adjacent to each other; in Sp2 one arm separates the SA and CA; in Sp3 two arms separate the SA and CA; and finally in Sp4, the SA and CA were opposite from each other; note that the start arm (in black) was 90° from either the SA or the CA (lower diagram), and the position of these baited arms alternated between left choice (LC) and right choice (RC) throughout trials. (D) The DNMP results are expressed as the average percentage of errors in all trials for each group (±SEM); note that WM and SC animals present significantly fewer errors than the IC group (***p<0.001, **p<0.01, *p<0.05) except in Sp4 were all groups perform well.

In independent groups of rats we tested both a behavioral (DNMP) and a neurophysiological (catFISH) measure of spatial pattern separation. The DNMP task [21] revealed behavioural evidence of spatial pattern separation improvement after the animals experience both swimming exercise and particularly contextual learning (Fig 1).

**Fig 2:**
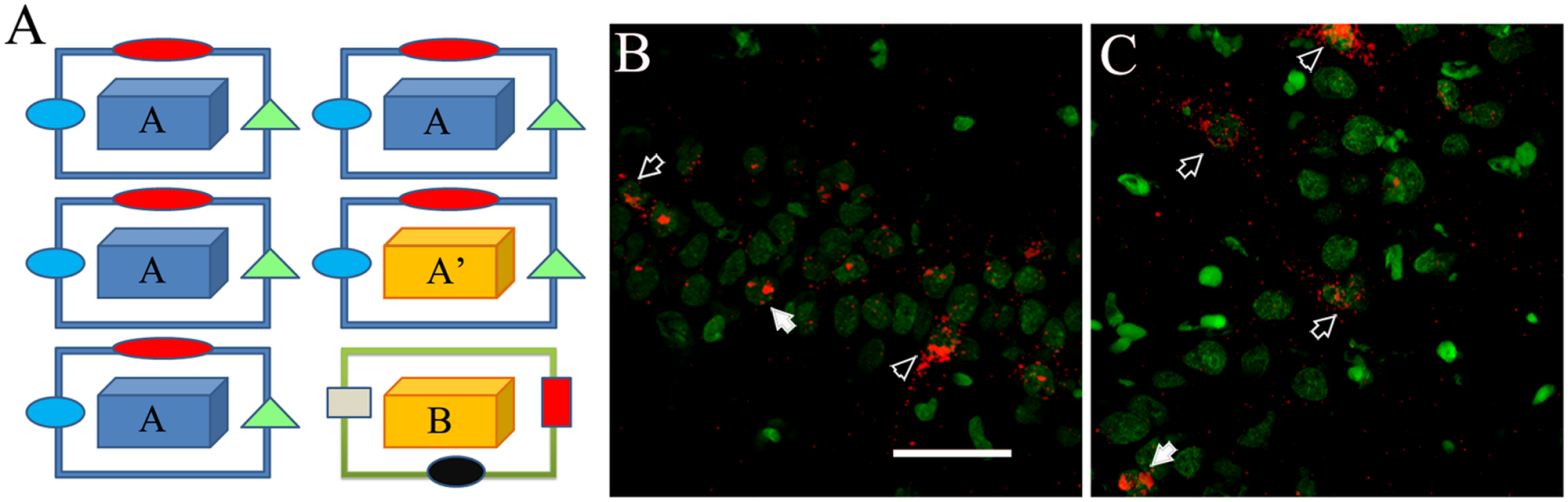
Exploration procedures and catFISH. (A) The animals explored the first environment for 5 min, and 25 min later they explored the second environment for 5 min in either the AA (same box same room), AA’ (different box same room), or AB (different box different room) condition. Animals were sacrificed immediately after the second 5 min exploration and their brains extracted, frozen and later process for FISH to detect *Arc* mRNA. Examples of CA1 (B) and CA3 (C) confocal images (1 filtered plane from the stack) stained for *Arc* mRNA detection and used to perform the catFISH analysis; in green is the nuclear counterstaining “Sytox” and in red is the *Arc* signal. Note that neuronal nuclei have texture, and their Sytox signal is dim, while glial cells look solid and bright. The *Arc* signal can be observed as 2 transcription foci inside the nuclei (solid arrows), or as cytoplasmic *Arc* staining surrounding the neuronal nuclei (open arrows), or as double Arc staining (arrowhead). The bar represents 50 μm.

The “catFISH” imaging method was done by detecting the immediate early gene *Arc* (or *Arg3*.*1*), and used as a neurophysiological measure of spatial pattern separation [22, 23]. *Arc* expression is used as a tag of neuronal activity [24], and we can distinguish its presence in the nuclei vs the cytoplasm, indicating recent or earlier neural activation, respectively. With this, we can distinguish the neural units activated by each of two spatial exploration experiences separated by a ~30-min interval, which we term epochs (Fig 2). With the similarity score from catFISH results we can determine, with one measurement, the extent of overlap between the ensembles recruited by each of the two independent behavioral epochs [23]. This provided a neurophysiological measure of spatial pattern separation [22, 23, 25 and 26] and support for the idea that a previous “spatial experience” improve it, as observed particularly in animals that underwent the WM, but also, the SC treatment.

Moreover, in the same animals used for catFISH, we also measured the area occupied by the synaptophysin staining in the hippocampal CA regions and found evidence of structural synaptic plasticity induced by the *“spatial experience”* (mainly in WM-treated and more subtly *in those treated as* SC animals). Interestingly, the increased area of synaptophysin staining correlated with the overlap measures obtained with catFISH, suggesting that this structural change may be related to some of the hippocampal information-processing improvements. Overall, the results indicate that a WM-training experience improves the behavioural and neurophysiological measures of spatial pattern separation, and suggest that the synaptic expansion induced by this behavioural experience may be related to the improvement of spatial information processing in the hippocampus.

## Methods

### Subjects

Four-month-old (±2 weeks, ~380grs ±43gr) male Wistar Rats obtained from the reproduction facilities at the Instituto de Neurobiología (UNAM) were housed individually in rat size acrylic boxes with rodents feeders cover, with wood shavings as bedding material, These boxes were located in the laboratory vivarium or animal room set to host up to 70 animals. The animal room is sound proof and water availability and disposal is in place. The animal room was kept on an inverted 12-h/12-h light/dark cycle with lights on at 9:00 p.m using an automated timer. The animal room also has a dim red light used for the experimenter’s convenience to perform the procedures carried out during the dark phase. Food (LabDiet) and water (filtered and UV treated) were provided *ad libitum* except during the delay non-matching to place (DNMP) task procedures, during which food consumption was restricted (see below). Animals were first habituated to the inverted light/dark cycle for 3 to 4 days, and then handled twice a day for 10 days prior to the behavioral procedures. The assignment of the animals to each group was done before all the experimental procedure begun, in a semi random manner, deliberately equilibrating the groups to include animals with equivalent weight and age measures in each experimental group.

The Bioethics Committee from the “Instituto de Neurobiología, de la Universidad Naciónal Autónoma de México”, which is ruled by Institutional Guidelines that stablish that the academic committee will decide and respond upon bioetical requests, issues and rules, such as the approval of animals protocols to be performed in this institute). This committe approved all protocols used in the present study (headed by Dra. Magdalena Giordano Noyola INEU/SA/CB/034 2010; and by Dr. Carmen Aceves Velasco INEU/SA/CB/034 2013). All procedures were performed in accordance with international ethical guidelines (as those approved by the NIH) for animal care and handling. All efforts were made to minimize the animals’ suffering.

### Behavioral Pre-treatments

#### Water maze overtraining (WM)

The Morris water maze task consisted in placing the rat into a cylindric tank of 1.5 m diameter that was filled to a height of 30 cm with water maintained at 23 ± 1°C. The platform was submerged 1 cm below the water surface. The room where the tank was located was sound proof, and the walls were covered with white foamy liners with visual clues (big geometric colored figures and drawings); the room was illuminated only by 3 sources of dim light located in 3 of the corners. In each trial the animal was released facing the nearest wall at each starting point. The animal was retrieved from the water tank 30 sec after it reached the platform, and then placed into a waiting cage for 60 sec. After this 60-sec wait, the animal was released again into the tank at the next starting point. When the animal did not find the platform within 60 sec, the experimenter guided the subject to the platform by hand and waited 30 sec with the animal on the platform before retrieving it. Training consisted of 5 identical daily sessions of 10 trials each. For each trial the animal was introduced into the pool at 1 of 10 different starting points (see [3] for more details). Each pair (i.e., 1–2; 3–4, etc.) had one starting point with a shorter distance to the target and the other with a longer distance to the target, but the average distance to the target was the same for each pair of starting points (i.e., 1–2; 3–4, etc.). The sequence designed for animals training was done in a semi random way that varied each day in which the experimenter that programmed such procedure only deliberately assigned an animal from each different group to the different time schedules throughout the duration of the procedure to spread all groups evenly across a time window of about 4 to 5 hours carried out between 10 hrs– 15 hrs. The rest of the behavioral procedures were done with the same rationale and time schedule.

Using a SmartSystem track recording device (San Diego Instruments, USA), we measured the latency to reach the target (in sec) as a measure of performance in the WM task. Since the average distance to the target is the same for each pair of trials, we used the average latency of each pair of trials (5 pair total) as a measure of WM performance for statistical analysis.

#### Swim control (SC)

Animals in this condition were placed in the same tank, located in same room used for the WM-training task. Each animal was released 10 times at each of the 10 different starting locations, simulating the 10 trials used in the WM-training procedure, but the escape platform and the spatial cues were removed. Each animal was allowed to swim for the average time expended by the WM-trained animals in each trial for each daily session, and the swimming session was terminated by the experimenter who placed the animal in a box located in an adjacent room. This procedure is slightly different from that used previously [2, 3]. The current procedure promotes more swimming, because swimming activity is stimulated each time the animal is released (10 total). Also, some of the room conditions, such as light coming from the adjacent recording room computers, was a remarkable difference we experience here from those previous experiments [2, 3]. However, the lighting and water conditions temperature and cleanness conditions were kept the same as in the WM-training task.

#### Intact control (IC)

Animals in this condition remained isolated in their home cages during the time the other animals underwent either the WM training or the SC procedure.

### Delayed non-Matching to Place (DNMP)

This task was performed in an 8-arm radial maze (RAM) apparatus (Lafayette Inst., IN USA) following a previously reported procedure [21]. The apparatus had dark metal floors and translucent acrylic walls (for details see Lafayette Inst., IN USA). A circular hole at the end of each arm gave the animal access to a plastic box with one open side from which the animal could retrieve the piece of food (1/2 of a piece of Kellogg’s Froot Loops); the other side was blocked by a metal grid, allowing the animal to smell the piece of food but not retrieve it. The day after the behavioral pre-treatment, all animals began a food-restriction schedule to maintain them at ~85% of their original body weight. A limited amount of food was provided once a day, and after a period of 5 to 6 days all animals reached this weight criterion. Seven days after the WM, SC, or IC pre-treatment, each animal was introduced into the RAM apparatus for one habituation session in which the animal was placed in the center octagon and allowed to retrieve a piece of food at the end of each arm; after retrieving all pieces of food the session ended. All behavioral testing occurred during the dark phase of the inverted light/dark cycle (lights on at 9:00 p.m). The experimenter who performed all the behavioural procedures was blind to all the previous experimental conditions.

The task began the day after habituation and consisted of two phases, the sample phase and the choice phase. The first arm through which the animal retrieved a piece of food is called the sample arm (SA), where food was available only during the sample phase; in the choice phase the food was retrieved from the choice arm (CA).

During the sample phase the animal retrieved the piece of food from the sample arm (SA), and only the start arm and the SA were open. Rats were removed from the maze and placed into a waiting box after retrieving the piece of food from the SA. After a 30–45-sec intervening interval during which the arms were cleaned with 6% acetic acid, the choice phase began. During the choice phase (Fig 1B), the rat was placed again in the start arm; 5 sec later the door was opened, and the rat could retrieve the piece of food from the choice arm (CA). Food was placed in the SA but was no longer available (grid closed). In the choice phase, the start arm, the SA, and the CA were open, and all other doors closed.

The choice arm and the sample arm are separated by 4 different angles (Sp1-45°; Sp2-90°, Sp3-135°; Sp4-180°). Separation 1 can be considered the most difficult challenge since the separation between the SA and the CA is minimal, as they are adjacent to each other. The task becomes easier with more separation between arms; separation 4 is the easiest, since the SA and the CA are in opposite positions. In each trial the CA alternates between a left or right choice relative to the SA, and the spatial location of both the SA and CA varies across trials (Fig 1C).

During the choice phase the animal needed to remember the previously visited arm and distinguish its location from that of the newly opened arm. A correct choice was noted when the rat entered the baited CA when appropriate (choice phase), and entries into other arms were considered an error; we also considered as an error when the animal entered the correct arm but returned to the center octagon before reaching the end of the arm (This counts represent less than 10% of the errors). All errors were used to compute the final results for statistical analysis. Note that the spatial locations of the SA and CA varied across trials, and also that the left/right disposition of the CA relative to the SA varied across trials (Fig 1B and 1C).

Each daily session consisted of 4 trials, and each trial consisted of one sample and one test phase. The 4 trials on any given day were done with the same separation, using the 4 different configurations randomly for each separation with the start being 90° from either the sample or choice arm. Over a 4-day period the separation was chosen semi-randomly for each animal, such that the sequence in which each separation day occur (Sp1, Sp2, Sp3 or Sp4) is not consecutive and apparently is not present in a pattern. But is important to note that the sequence used in each animal was deliberately different, but the chosen days were selected with the same criteria. All animals experienced a total of 4 sessions with each separation, that is, 16 trials for each separation over a 16-day period for a total of 64 trials per animal. We used the percentage of errors for all trials as the dependent variable for statistical analysis and compared the IC, SC, and WM groups for their ability to distinguish between each separation.

### Double Spatial Exploration for catFISH analysis

In an independent group of animals, this behavioral procedure was carried out in two different but adjacent rooms located 3.5 m from the animal colony room. Spatial exploration was carried out in an open field box (aprox 80 x 80) placed in a room, and the animals were handled for “forced exploration” for two “epocs”.

These two “epocs” occur in 3 different forms:

(AA) same room, same box

(AA’) same room, different box

(AB) different room, different box

We refer to these 3 conditions as the double exploration conditions. For details, each different box (A or B) was partitioned into 9 quadrants. The procedure consisted in placing the rat into quadrant number 1; then every 15 sec the animal was “moved” to the center of the next quadrant “by the experimenter”, and after 5 min the animal had visited each of the 9 sectors of the box 2 or 3 times each, consistently. This procedure is considered forced exploration, and it is used to minimize the variability in the number of Arc-expressing cells among animals, but it induces Arc expression in the same proportion of neurons as free exploration [23]. Also, it minimizes the total exploration variability, since animal’s release stimulates exploration. Laboratory observations and previous work [23] suggest that the amount of actual exploration of the animals is similar among conditions (AA, AA’, AB).

After the first 5-min exploration, the rat was returned to its home cage for 25 min before beginning the second 5-min exploration, either in A again, or in A’, or in B. (Fig 2A).

*Exploration A* was done in an open, 70-cm square acrylic box with 20-cm-high walls that was located in room 1, which was 1.7 m x 2.3 m and had walls covered with sound-isolation materials and 2 posters as spatial cues.

*Exploration A’* was done in a rectangular 65 cm x 75 cm open acrylic box with 20-cm-high walls located in room 1.

*Exploration B* was done in a 65-cm x 75-cm open acrylic box with 20-cm-high walls; it was located in room 2, which had dimensions of 2.17 m x 2.3 m; the walls were covered with sound-isolation materials and several colorful, foamy-paper figures as spatial cues.

Cage controls (CC): this condition was used to determine the basal levels of *Arc* expression, and the animals from this group remained undisturbed in their home cage and were sacrificed at times distributed throughout the time the rest of the animals underwent the double spatial exploration experience.

### Compartamental Analysis of temporal activity using Fluorescence “In Situ” Hybridization (catFISH)

#### Brain extraction and cryosectioning

After the last spatial exploration (or CC condition) rats were sacrificed by live decapitation, and their brains were quickly extracted (< 3 min) and frozen (see [23]). Brain hemi-sections including the whole dorsal hippocampus were obtained; up to eight brain hemi-sections were placed into a mold, and a block was made using Tissue-Tek OCT compound (Miles, Elkhart, IN). Each block was cryo-sectioned into 20-μM-thick coronal slice sections (Leica 1850, Leica Biosystems, Nussloch Germany) that were placed on a Superfrost Plus slides (VWR International).

#### Fluorescence In Situ hybridization (FISH)

The slides on which the 20-μm brain sections were mounted were immersed in 4% buffered paraformaldehyde for tissue fixation. The tissue was then washed in 2XSSC (Sigma, International) and treated with 0.5% acetic anhydride/1.5% triethanolamine (Sigma). After washing, the slides were immersed in 1:1 acetone/methanol solution, and then incubated in 1× pre-hybridization buffer (Sigma) for 30 min at room temperature. The *Arc* antisense riboprobe was synthesized in our laboratory, using digoxigenin-labeled nucleotides, from a plasmid kindly given by Dr. Paul Worley’s lab. The *Arc* riboprobe was diluted into 100 μL of hybridization buffer (Amersham), denatured by heating at 95°C for 5 min, and then chilled on ice until it was added to each slide (100 ng riboprobe per slide section). Cover slips were placed on each slide, which was then placed in a plastic chamber and incubated in an oven (Binder, Tuttlingen Germany) at 56°C for 16 h. After the probe incubation period, the slides were washed several times in 2xSSC, then in 2×SSC with RNase A (10 μg/mL) at 37°C to eliminate non-hybridized probe, and finally in 0.5 × SSC at 56°C. Endogenous peroxidase activity was quenched with 2% H_2_O_2_ in Tris-buffered saline (TBS, Sigma), and then the slides were blocked (TSA Blocking Buffer, Perkin Elmer, MA USA) prior to an overnight incubation with anti-digoxigenin (HRP)-antibody conjugate (Roche, Hertfordshire, UK. Cat# 11093274910, RRID: AB_514497) at 4°C. The specificity of this antibody was previously confirmed using appropriate control procedures, which are performed routinely by those performing the histological procedures in our laboratory. Slides were then washed in TBS containing 0.05% Tween-20, and the anti-digoxigenin antibody was revealed by using the Cy3 TSA system (Perkin Elmer, MA USA, diluted 1:75). Slides were then incubated with SYTOX green (Invitrogen, International) diluted 1:2000 in TBS to stain the cell nuclei. Finally, Vectashield anti-fade medium (Vector, Burlington, CA USA) was applied to each slide, and it was coverslipped and sealed with nail polish (for further details see [23]).

#### Image acquisition and analysis

**Fig 3:**
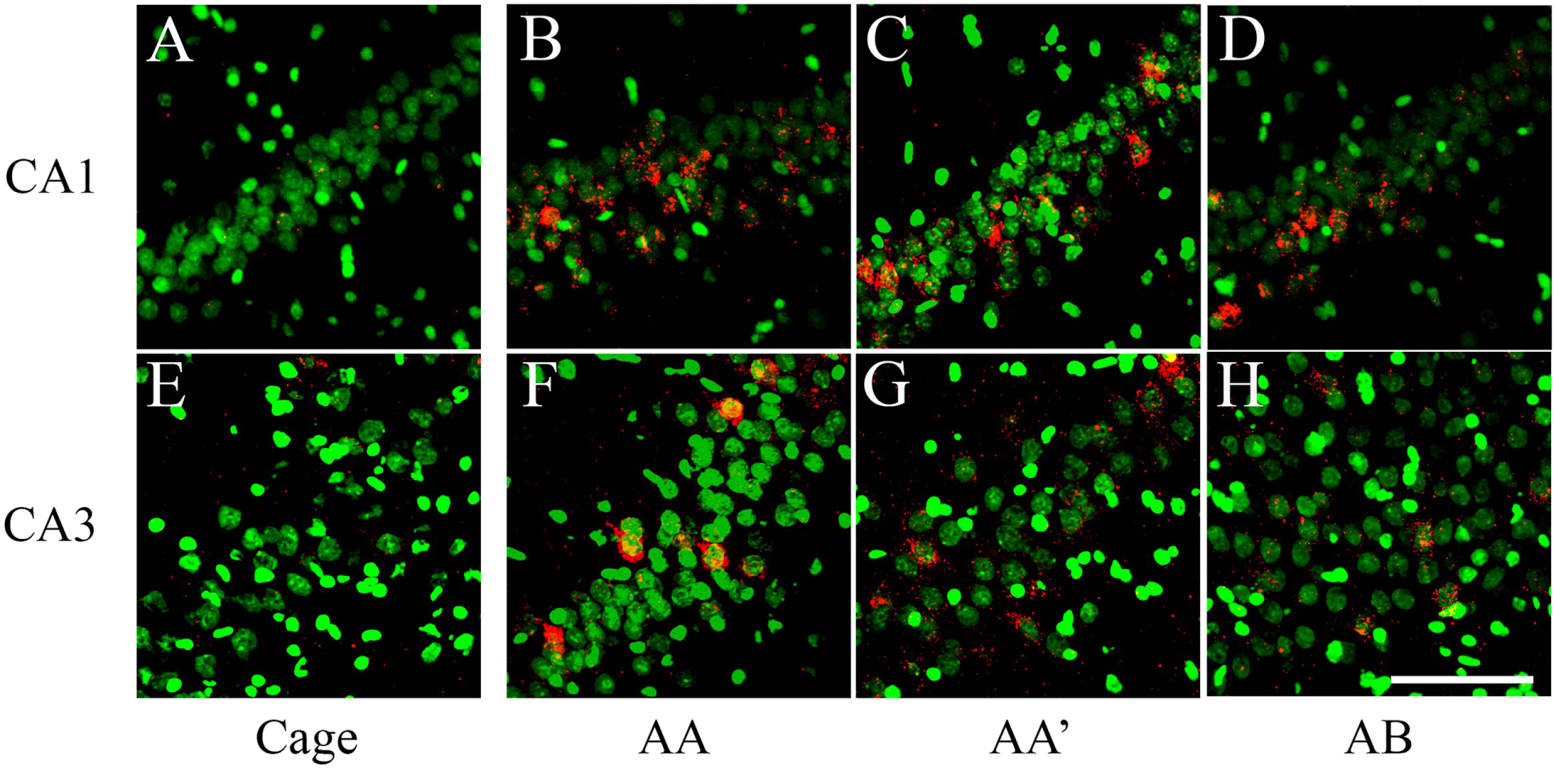
catFISH images from CA1 and CA3 from the different exploration procedures. Dorsal hippocampal tissue stained with fluorescent *in situ* hybridization (FISH) for *Arc* mRNA (in Red) revealed with Cy3 (Promega kit see Methods). Images were taken with the 40x/1.3 NA objective using a Zeiss LSM 510 or Zeiss LSM 710 confocal system with 2 different lasers either set to excite Cy3 (*Arc*) or SITOX-Green (Nuclear counterstaining in Green). All images were process with Image J and 40–55% of the stack was collapsed in 1 single image (using Z Project). Images then went through the median filter, using the exact same parameters for all (0.8 pixels). From A to D, the images were taken from the CA1 hippocampal network and from E to G, from the CA3 network. Images A and E were taken from a Cage control animal; B and F from an animal that underwent the AA double exploration; C and G from an animal that underwent the AA’ double exploration condition; and D and H from an animal that underwent the AB double exploration condition. Calibration bar (lower right) represents 100 μm.

Image stacks from 4–6 optimally *Arc*-FISH-stained slides were acquired using a Zeiss LSM 510 confocal microscope (Zeiss, Mexico) with a 40×/1.3 NA oil immersion objective, using the 543-nm helium/neon laser to excite the CY3 signal, and the 488-nm argon laser to excite the Sytox green signal. Routinely, the confocal settings for the CY3 Arc signal were optimized to detect only the intranuclear and cytoplasmic staining, minimizing the noise by adjusting the laser power, the amplifier, and offset accordingly. These parameters were established on a tissue section from a cage control animal and were kept constant for imaging all other brain sections on the same slide. The pinhole, Z-sectioning interval, and gain settings were kept constant for all the imaging. About 34–50 optical Z-sections of ~0.3-μm thickness were obtained from the 20-μm-thick tissue for each CA1 and CA3 image stack (Fig 2B and 2C; Fig 3). Four image stacks were obtained from each brain region (CA1 and CA3) for a total of 8 images per section on the slide and, as mentioned before, a total of 4 to 6 optimally stained slides per block were imaged.

Confocal image stacks were analyzed using the Image J analysis software (Wayne Rasband, NIH). The experimenter who performed the analysis was blind to all the experimental conditions. All images were pre-processed with a median filter for noise reduction before manual-count analysis by human eye. The Sytox green nuclear counterstaining signal (which appears green in the images; Fig 2B and 2C; Fig 3) was used to identify the neuronal nuclei that were included in the analysis, while the glial nuclei were not included. This nuclear classification was previously validated using immunohistochemistry for neuronal and glial makers [27]. To minimize sampling errors and stereological concerns, an optical dissector technique was used, in which only neuron-like cells found in the middle 30% of the stacks were included in the analysis [23, 24]. This technique prevents the minor variations in cell volume from influencing sampling frequency and minimizes sampling errors and stereological concerns attributable to partial cells. An average of 1100 neurons per animal in CA1 and 920 neurons per animal in CA3 were included in the analysis.

**Fig 4:**
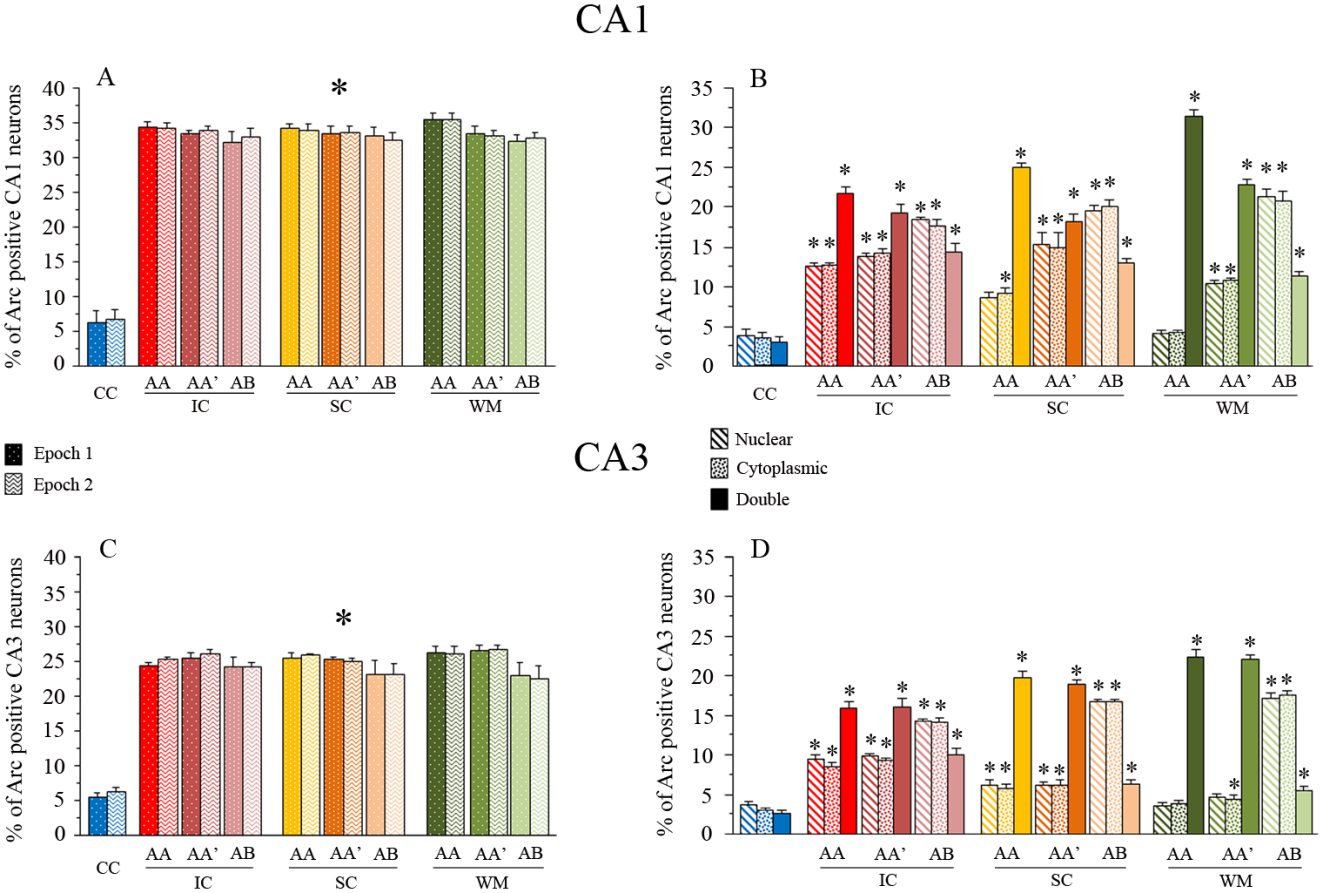
Raw catFISH results. The bar graphs (in A and C) show the percentage of *Arc*-expressing cells in the CA1 (A) and CA3 (C) networks in each epoch. The solid bars represent the proportion of active cells for epoch 1, and the lighter, textured bars represent the proportion of active cells for epoch 2 (*p<0.01 Bonferroni posthoc analysis for all exploration groups vs. the cage control). The percentage of Arc-expressing cells for each classification is shown in B and D. Each bar represents the proportion of nuclear (Diagonal line pattern), cytoplasmic (Dots), or double (Solid) *Arc* mRNA staining. Note that the proportion of double activated cells in both CA1 and CA3 after the AA exploration condition is larger than other classifications (i.e., nuclear and cytoplasmic), which is particularly clear in the WM-treated animals (*p<0.01 Bonferroni posthoc analysis vs. the cage control).

Once neurons were identified, they were classified according to their cytoplasmic and nuclear *Arc* mRNA staining, detected with the CY3 signal (Fig 2B and 2C; Fig 3). Neurons whose nuclei were > 60% surrounded by CY3 signal in 4 or more Z-section planes were classified as cytoplasmic Arc-positive cells (Fig 2B and 2C; Fig 3). Neurons with two intense CY3 nuclear foci visible across 3 or more Z-section planes were classified as nuclear Arc-positive cells, and those that fulfilled both criteria were classified as double-activated cells. Cells that did not fulfil any of the previous criteria were considered Arc negative. The analysis yields the proportion of *Arc*-mRNA positive neurons (from each classification) out of the total population of neurons included in the analysis (Fig 4).

#### Similarity score calculation

This score takes into account the four possible cell-staining classifications: Negative (Neg), Arc intra-nuclear foci (Arc-Nuc), Arc cytoplasmic (Arc-Cyt), and Arc double (Arc-Dob) and reduces them to a single value according to the following procedure:

Arc-Nuc = Number of cells classified as: *Arc* nuclear

Arc-Cyt = Number of cells classified as: *Arc* cytoplasmic

Arc-Dob = Number of cells classified as: *Arc* double

Total-Cells = Number of cells classified as: Neg + Arc-Nuc +Arc-Cyt + Arc-Dob.

Epoch 1 = (Arc-Cyt+Arc-Dob)/Total-Cells)

Epoch 2 = (Arc-Nuc+Arc-Dob)/Total-Cells)

leastEpoch = Lowest value “Epoch 1 or Epoch 2”

p(E1E2) = Epoch 1 * Epoch 2

diff(E1E2) = (Arc-Dob/Total-Cells)-p(E1E2)

SiSc = diff(E1E2) /(leastEpoch—p(E1E2))

**Fig 5:**
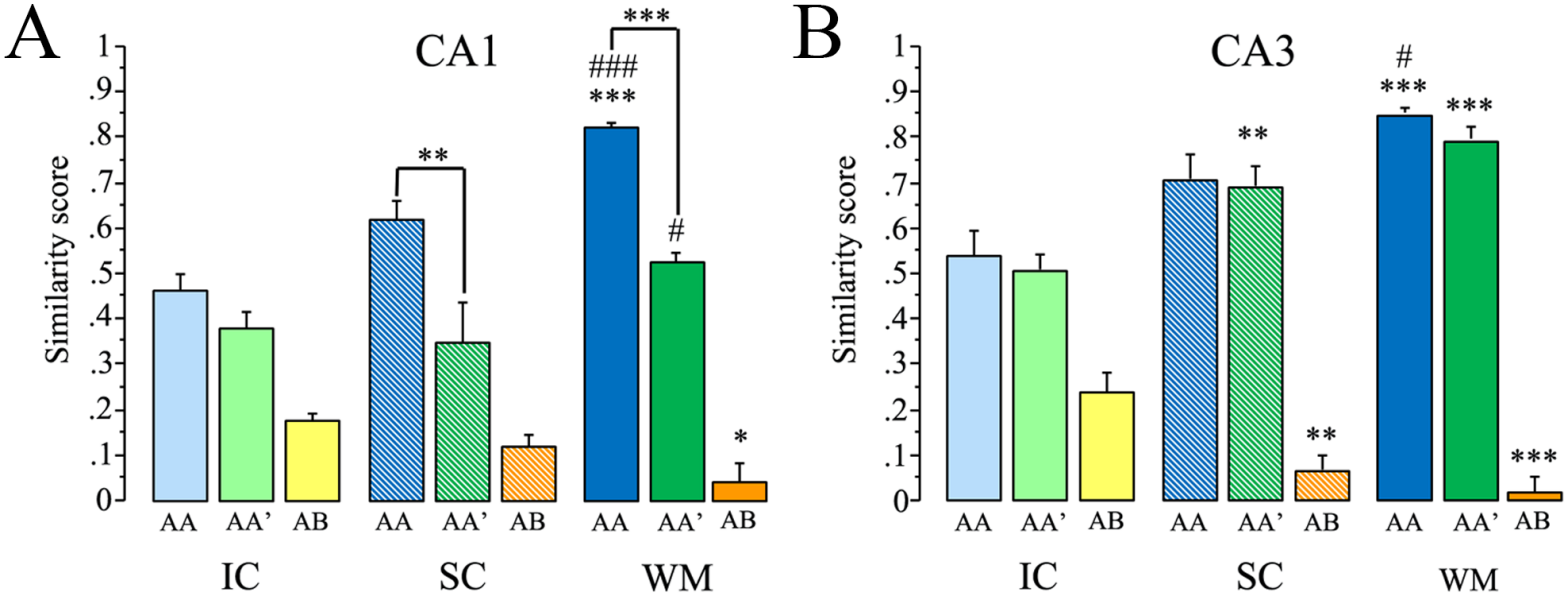
The similarity score (SiSc) results from catFISH. This measure takes into account the 3 Arc staining classifications and expresses it in a single value. This value (±SEM) represents the degree of overlap between the two recruited ensemble in the CA1 (A) and CA3 (B) networks from animals exposed to the AA, AA’, and AB double exploration conditions after being pre-treated as either intact controls (IC), swimming controls (SC), or water maze trained (WM). Bonferroni *post hoc* analysis of the WM group vs. its respective IC ***p<0.001, **p<0.01, *p<0.05; or vs. its respective SC (###p<0.001, #p<0.05), and the important intragroup differences are shown with lines (***p<0.001, **p<0.01).

A value close to 1 represents a single neuronal population faithfully activated in both behavioral epochs, whereas a value close to 0 indicates that two, statistically independent cell populations were activated during the two epochs [Fig 5].

### Synaptophysin/Map2 staining

#### Double immunoshistochemistry for synaptophysin and Map2

Coronal brain sections (20 μm) containing the dorsal hippocampus mounted on superfrost (VWR) slides adjacent to those used for catFISH were fixed with 2% buffered paraformaldehyde, quenched in 0.6% H_2_O_2_ in TBS for 15 min, and blocked with TSA blocking buffer (PerkinElmer, MA USA) at room temperature for 30 min. The sections were then incubated overnight at 4°C with monoclonal mouse anti-synaptophysin antibody (Sigma Cat# S5768, RRID: AB_477523) diluted 1:250 in TSA blocking buffer. The specificity of this and the other antibodies used here were previously verified using appropriate control procedures. After incubation, the slides were washed with TBS, pH 7.5, containing 0.05% Tween-20 and later with TBS alone. After washing, the slides were incubated in the secondary antibody (biotinylated goat-anti mouse, Vector Cat# BA-9200, RRID: NA, 1:500 in TSA blocking buffer) for 2 h at room temperature, washed, and then incubated in the Avidin-Biotin (AB) amplification reagent (Vector Labs, CA USA) for 45 min. After washing with TBS, the slides were incubated for 45 min with the CY3 fluorophore from the TSA system (diluted 1:75 in amplification buffer from the Perkin Elmer kit, MA USA). Slides were then treated with mouse-on-mouse (Vector Labs Cat# MKB-2213, CA USA) following the manufacturer’s instructions for double detection with 2 antibodies raised in mouse. After this, the slides were washed and blocked with TSA blocking buffer for 20 min. We then incubated them overnight at 4°C with the monoclonal mouse anti-MAP-2 antibody (1:100, Millipore Cat# AB5622, RRID: AB_11213363, MA USA). After incubation, the sections were washed in TBS, incubated in the AB blocking kit (Vector Labs, CA USA), washed again, and then incubated with the secondary antibody (biotinylated goat-anti mouse, Vector Cat# BA-9200, RRID: NA, 1:500 in TSA blocking buffer) for 2 h at room temperature. After washing, the slides were incubated in the AB amplification kit (Vector Labs, CA USA) for 45 min, washed again, and finally incubated with 1:100 FITC fluorophore from the TSA system kit (Perkin Elmer). After this final detection, slides were counterstained with DAPI. VectaShield mounting medium was applied and cover slips were placed over the slides. All antibodies were validated for specificity, using appropriate controls such as incubation without the primary or secondary antibodies, performed by the experimenter(s) in charge of the histological procedures. Finally the slides were sealed with nail polish and stored in the dark at 4°C until used for image acquisition.

#### Image acquisition and analysis of synaptophysin- and Map2-stained sections

MosaiX images were obtained with the APOTOME system equipped with an oil-immersed 25X/0.80NA Plan-Apochromat objective. The MosaiX module (Carl Zeiss, International) was used to construct one image mosaic or montage containing a total of 30 images with which we captured the entire CA3 and CA1 hippocampal regions from each coronal brain section on the slide. A total of 7 to 8 montage images (from 7 to 8 slides) per animal were obtained. The imaging parameters were set in one IC animal from each slide, and were optimized to highlight both Map2 (FITC) and synaptophysin (Cy3) staining found in the CA3 *SL*, minimizing the background for these signals. Once these parameters were set, they remained constant for the rest of the slide.

**Fig 6:**
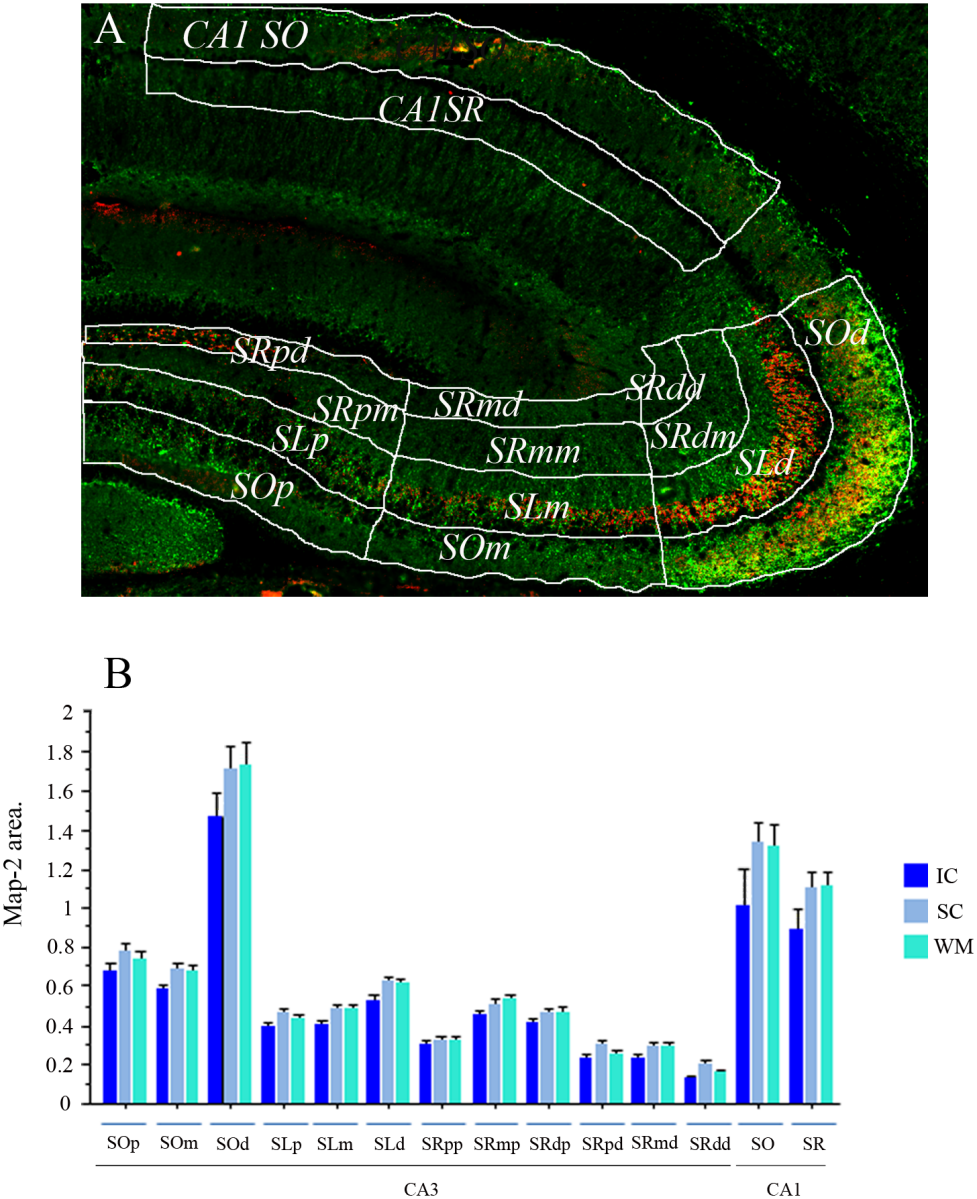
Synaptophysin/Map2 segmentation and Map2 staining area analysis. (A) A representative synaptophysin (Red) / Map2 (Green) stained image is shown with the drawings that defined the different hippocampal dendritic segments regions of interest (hippocampal segments ROIs). These hippocampal segments ROIs included the CA3 *stratum oriens*, divided into 3 regions based on their proximity to the dentate gyrus (DG): *stratum oriens distal* (*SOd*), *stratum oriens medial* (*SOm*), and *stratum oriens proximal* (*SOp*); the CA3 *stratum lucidum* was also divided into 3 regions, the *stratum lucidum distal* (*SLd*), *stratum lucidum medial* (*SLm*), and *stratum lucidum proximal* (*SLp*). The CA3 *stratum radiatum* was divided into 6 regions, depending on their proximity to the DG and to the pyramidal cell soma: *stratum radiatum distal medial* (*SRdm*), *stratum radiatum medial medial* (*SRmm*), *stratum radiatum proximal medial* (*SRpm*), *stratum radiatum distal distal* (*SRdd*), *stratum radiatum medial distal* (*SRmd*), and *stratum radiatum proximal distal* (*SRpd*). Finally, the last 2 ROIs correspond to the CA1 *stratum oriens* (CA1 *SO*) and the CA1 *stratum radiatum* (CA1 *SR*). In the bar graph (B) the Map2-stained area expressed in pixels is shown for each hippocampal segment ROI. It is important to emphasise that No significant differences were found among groups (IC, SC, and WM) in the Map2-stained area used for the synaptophysin analysis.

Analysis of the immunofluorescence for synaptophysin/Map2 staining was performed using ImageJ software (Freeware NIH USA). All images were pre-processed with the median filter for noise reduction. Then, each image was segmented into 14 different regions of interest (ROIs). The ROIs were drawn to segment different portions of the CA3 and CA1 dendritic regions (Fig 6A). In CA3, we distinguished between the *stratum oriens* and *stratum lucidum*, and between proximal and distal portions of the *stratum radiatum* based on their proximity to the CA3 pyramidal cell soma. The CA3 region was also divided into 3 regions (distal, medial, and proximal) based on their proximity to the DG granular layer (12 ROIs total in CA3). Finally, we divided the CA1 region between the *stratum oriens* (CA1 *SO*) and *stratum radiatum* (CA1 *SR*). It is possible that the 2 ROIs selected in the CA1 region may contain a portion of CA2. These ROIs are shown in Fig 6A and listed in the legend. Once the ROIs were drawn, we established an optical density threshold for each signal (CY3 and FITC) using the hippocampus MosaiX image from the IC on the same slide. We then measured the area occupied by each signal in each ROI and performed the same procedure on each image of the slide, keeping the same threshold parameters and following the same procedure. The data we obtained were the areas occupied by each signal expressed in pixels. It is important to clarify that Map2 signal was used as a guide to draw an ROI of the area surrounding the Map2 staining edges, defined with a threshold, and about a 10% enlargement to cover the possible area were the synaptophysin staining should be. For synaptophysin, the area measured was the actual staining area detected; the thresholds for both Map2 and Synaptophysin were systematically set in a cage control tissue from each slide and kept constant for the rest of the slide analysis. Then, the measures used for analysis was the area of synaptophysin staining divided by the ROI area defined by the Map2 staining x 100. And note that no differences were found in any ROI in the MAP2 defined area between groups (Fig 6B).

**Fig 7:**
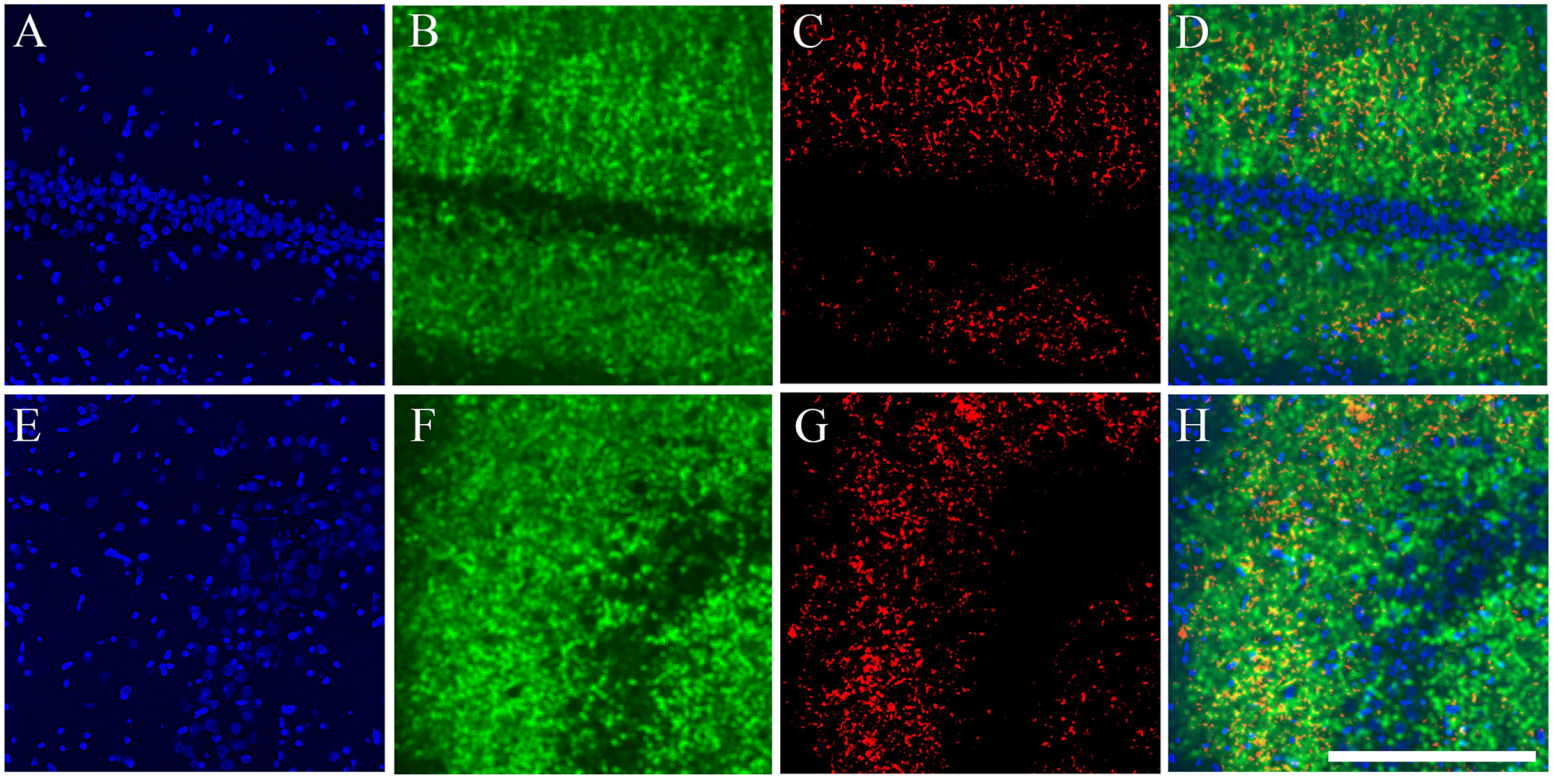
Synaptophysin/Map2 Split Channel and Higher Magnification Images. Immonostained tissue for synaptophysin and Map2 imaged with the Zeiss ApoTome™ system, equipped with a motorized stage which allows the acquisition of montage or mosaic images with the MosaiX software. Images were taken with the 25X/0.8 NA objective. One Zeiss LSM file containing the MosaiX montage was open using imageJ software and the color channels were separated (split channels). Then each channel went through the median filter and assigned its representative color. The nuclear counterstaining DAPI is shown in blue (A for CA1 and E for CA3). Map 2 is shown in Green (B for CA1 and F for CA3) and synaptophysin is shown in RED (C for CA1 and G for CA3). The merge colors image is shown in D for CA1 and H for CA3. The magnification shown here was done by only trimming the montage image and by applying a regular zoom-in using adobe photoshop. This is a proper example of the image resolution the experimenter had available for analysis (He can perform the same simple zoom-in magnification with the same results), and was obtained from a similar montage image as those shown in Figs 6 and 8. Calibration bar (lower right) represents 200 μm.

Synaptophysin staining is observed particularly clear in the hippocampal region CA3 but is also clear with the proper magnification in the CA1 region (Fig 7C and 7G), this may be because of the prominent size of the MF boutons. It is also ubiquitously observed throughout the brain and the rest of the hippocampus, with different signal qualities. Previous reports in our field used synaptophysin immunostaining to detect MF expansion after behavioural experience [4, 28]. We also used Map-2 staining since this protein is constitutively express in dendrites of hippocampal pyramidal cells and for that reason it can be used to reveal the dendritic shape in relation to the synaptophysin stained area. This procedure provides a correction parameter for anatomical variations concerns due to the histological procedures, such a shrinkage or flattening of the tissue. For this reason it is more accurate to express a ration of synaptophysin stained area on the Map-2 selected ROI area.

It is important to acknowledge that synaptophysin is a protein present in synaptic vesicles. For this reason with this staining we can only detect part of a pre-synaptic structure, more detailed experiments should be done to visualize and measure the whole synaptic structure (i.e. pre and post synaptic).

### Statistical analysis

Group differences were analyzed by a one-way ANOVA followed by a Fisher or Bonferroni *post hoc* test where appropriate, also a 2 way ANOVA was applied were appropriate. A repeated measure ANOVA was also performed for the behavioral results, and Pearson’s correlation analysis was used on the SiSc and the synaptophysin/Map2 area. A paired T test was used to compare CA1 and CA3 Arc-positive neurons after exploration.

## Results

### Morris water maze overtraining improves the performance in a DNMP task

Since the DNMP task was previously used to measure spatial pattern separation and WM training induces mossy fiber expansion, we test the hypothesis that WM training inducing MF expansion could improve the performance of animals in the DNMP task.

Training in the WM consisted of 5 daily sessions of 10 trials each, and the latency to reach the target in each pair of trials (5 pairs) was used for statistical analysis (see Methods). The results revealed a significant learning curve on each day (Repeated measures ANOVA: day 1, F 8,4 = 6.61 p<0.001; day 2, F 8,4 = 12.679 p<0.001; day 3, F 8,4 = 7.481 p<0.001; day 4, F 8,4 = 3.799 p = 0.0123; and day 5, F 8,4 = 3.028 p = 0.0317), indicating that latency gradually decreased on subsequent trials each day. Note, however, that on the last two days (4th and 5th), the animals reached a performance plateau (Fig 1A; S1 File); this kind of performance was previously considered as overtraining [3].

Seven days later, the WM animals (WM n = 10), animals treated as swimming controls (SC n = 11), and an intact control group (IC n = 13) were trained in a delay non-matching to place (DNMP) task designed to test spatial pattern separation [21]. The number of animals is similar to that used in a previous report [21]. In the present experiment, the DNMP tast was carried out in two phases (with half the animal sample in each phase) separated by a 6 weeks interval, no clear behavioral differences in the animal’s performance were detected between phases.

This task tests the ability of rats to choose between two arms with different spatial separations.

The results revealed significant differences between groups in the number of errors in Sp1 (one-way ANOVA: F 2,31 = 8.45 p<0.01), Sp2 (F 2,31 = 5.63 p<0.01), and Sp3 (F 2,31 = 3.36 p<0.05). The *post hoc* Fisher analysis revealed highly significant differences between WM and IC animals in both Sp1 and Sp2 (p<0.01) and also significant differences in Sp3 (p<0.03). The SC animals and also WM showed significantly fewer errors than IC animals in these 3 separations (p<0.05 and p<0.01, respectively). No differences were found between the SC and WM groups. Overall, the behavior of both SC and WM animals indicated improved spatial pattern separation (Fig 1D: S2 File).

When the number of errors across the different separations were compared (repeated measures ANOVA), we found significant differences between groups (F 2,31 = 5.528 p<0.01) and between separations (F 3,6 = 4.754 p<0.01). The *post hoc* Fisher analysis revealed a significantly higher number of errors in Sp1 and Sp2 when compared to both Sp3 and Sp4 (p<0.05), indicating that errors were made mainly in the hardest challenges (Sp1 and Sp2). For the group comparisons the *post hoc* Fisher analysis revealed significant differences between the WM and SC groups compared to the IC group (p<0.01 and p<0.01, respectively), confirming that SC and particularly WM-treated animals perform better than the IC group.

When this analysis was done with the split by groups option, which allowed us to perform the repeated measures ANOVA in each individual group (WM, SC, and IC), significant differences between Sp1 and Sp2 vs Sp3 and Sp4 were found only in the IC group (F 3,12 = 6.37 p<0.01), indicating that this group did not distinguish well between closely spaced arms.

### Cellular-compartment analysis of temporal activity using fluorescent in-situ hybridization (catFISH) for the immediate early gene Arc

Vazdarjanova and Guzowski [23] had previously shown results obtained with catFISH and the SiSc to measure the degree of overlap in the ensembles recruited by a double spatial exploration experience of different contexts (AB) that directly or indirectly [18] reflects spatial pattern separation. With that in mind we decided to measure with catFISH and the SiSc results, obtained after 3 different double spatial exploration conditions (AA, AA’ and AB), the degree of overlap between the recruited ensembles as a measure that indicates, directly or indirectly, spatial pattern separation. A good bet given the DNMP evidence indicating that previous *“spatial experience”* improves spatial pattern separation. We then studied the neurophysiological correlates of this behavioral improvement by using the compartamental analysis of temporal activity with fluorescence in situ hybridization (catFISH) imaging method [24].

In an independent group of animals (n = 42), we performed catFISH in the CA1 and CA3 hippocampal networks. Here again, the animals were pre-treated in either the WM task (n = 13), the SC condition (n = 13), or as IC (n = 16). Seven days later, all the animals were exposed to a double spatial exploration experience for catFISH [24], which consists of two 5-min spatial-exploration epochs separated by an interval of 25 min, with 3 possible configurations: The AA groups (n = 4 per group, total n = 12) experienced a double spatial exploration in the same box and the same room; the AA’ groups (n = 4 per group, total n = 12) experienced a double spatial exploration of two different boxes located in the *same room*; and the AB groups (n = 4 per group, total n = 12) underwent a double exploration of two different boxes located in different rooms (Fig 2A). The rest of the animals (n = 6) were used as cage controls (CC) for the catFISH analysis, providing a negative control for *Arc* mRNA expression (Fig 3A and 3E). The number of animals used in catFISH experiments [23, 24] can be low since the statistical validity of the catFISH analysis is based more upon the number of neural units included in the analysis, a similar rationale as that use in electrophysiological measure, whit greater numbers (An average of 1100 neurons per animal in CA1 and 920 neurons per animal in CA3 were included in the analysis). In the present experiments, the catFISH study was also done in duplication with about 3 animals per double exploration condition and pre-treatment, the final N was reached after selecting the blocks from each catFISH run based in the assessment, performed by a experimenter blind to the experimental conditions, of optimal staining and preservation conditions of the tissue sections in the block.

Note that the two boxes used for the double exploration conditions (in AA’ and in AB) differed only slightly in shape and that the most salient feature that distinguished the explorations was the room (see Methods).

The raw catFISH results (Fig 4) revealed that in the CA1 hippocampal network each spatial-exploration epoch induced *Arc* mRNA expression in ~33% of the pyramidal neuron population (Fig 4A; S3 File). A one-way ANOVA of the total Arc-expressing cells in each epoch revealed significant differences among groups (Epoch 1: F _9, 32_ = 66.42 p<0.01; Epoch 2: F _9, 32_ = 83.56 p<0.01), and the respective Bonferroni *post hoc* analyses revealed that all groups showed a significantly higher percentage of neurons positive for *Arc* mRNA (all classifications) than the cage control group (p<0.001) for all exploration groups, and no differences in the proportion of *Arc*-expressing cells were found among the groups of animals that underwent the double spatial exploration experience. In the CA3 network each behavioral epoch induced *Arc* mRNA expression in ~25% of the pyramidal neuron population (Fig 4C; S4 File); similarly, the one-way ANOVA performed on the percentage of CA3 neurons expressing *Arc* mRNA in each epoch revealed significant differences among groups (Epoch 1: F _9, 32_ = 142.89 p<0.01; Epoch 2: F _9, 32_ = 148.27 p<0.01), and the respective Bonferroni *post hoc* analyses revealed that all groups showed a significantly higher percentage of neurons positive for *Arc* mRNA (all classifications) than the cage control group (p<0.001), while differences among the exploration groups were not detected. These results indicate that each spatial exploration epoch stimulates neural activity (significantly more Arc-expressing neurons above CC levels) in both the CA1 and CA3 hippocampal networks and that this activity results in *Arc* mRNA expression in a percentage of neurons that does not differ among exploration groups or between epochs. It is important to note, however, that the same exploration experiences recruit different proportions of neurons in CA1 than CA3 networks (t = 35, -22.903, p<0.05).

By distinguishing between nuclear, cytoplasmic, and double *Arc* mRNA staining, we were able to distinguish between neurons activated only by the last exploration (Nuclear staining), neurons activated only by the first exploration (Cytoplasmic staining), and neurons activated by both explorations (Nuclear and Cytoplasmic staining).

We observed significant differences in the proportion of *Arc* positive neurons between the double exploration conditions in both the CA1 (Fig 2B) (Nuclear: F _9, 32_ = 71.81 p<0.01; Cytoplasmic: F _9, 32_ = 50.5 p<0.01; double: F _9, 32_ = 106.313 p<0.01) and the CA3 (Fig 2D) (Nuclear: F _9, 32_ = 112.19 p<0.01; Cytoplasmic: F _9, 32_ = 157.05 p<0.01; double: F _9, 32_ = 119.19 p<0.01) hippocampal networks.

These raw catFISH results appear to show that in both the CA1 and CA3 networks, the WM- and SC-pre-treated animals tend to use more overlapping ensembles after the AA double exploration condition and more independent (less overlapping) ensembles in the AB double exploration condition, in contrast to the intact control animals. But a more eloquent analysis required further data processing, so we used the similarity score (SiSc) measure, which is obtained by reducing the four cell-staining parameters (negative, *Arc*-nuclear [second epoch only], *Arc*-cytoplasmic [first epoch only], and *Arc*-double [both epochs]) into a single value; a SiSc value close to 0 represents the selection of two statistically independent ensembles of neurons in each epoch, while a value close to 1 represents the selection of the same ensemble of neurons in each epoch [23]. For the SiSc analysis we excluded the cage-control animals, since they were used only to determine the basal levels of *Arc* mRNA expression and to establish that the observed proportion of *Arc* mRNA-expressing cells resulted from the behavioral experience.

The SiSc in the CA1 network differed significantly among the different groups (F _8, 27_ = 62.16 p<0.01). Bonferroni *post hoc* analysis revealed that when the animals were exposed to the AB condition, the SiSc of the CA1 network from WM-treated animals was significantly lower than that obtained in IC animals (p<0.05); similarly, after this double exploration condition (AB) the SiSc obtained in the hippocampal region CA1 from SC animals did not differ from that of the IC group, indicating that only WM animals present the lowest degree of overlap after the AB condition. This can be interpreted as an improved spatial pattern separation in the CA1 network of WM-treated animals (Fig 5A; S3 File).

Interestingly, when WM animals were exposed to the AA’ condition, their SiSc in CA1 was significantly lower than that obtained in the AA condition (p<0.001), indicating that in response to gradual changes in the environment, the CA1 network of WM animals reacts by recruiting a slightly different ensemble, and this can be interpreted as partial pattern separation in the CA1 network of WM animals. It is important to point out that also in the CA1 region from SC animals the SiSc was significantly lower after the AA’ double exploration than after the AA condition (p<0.001) Importantly, these differences in the SiSc obtained in the CA1 between the AA and AA’ conditions were not found in the IC group. These results indicates that the CA1 network reacts to the altered input pattern by creating a similarly altered output representation, and this may imply partial remapping [26] or partial spatial pattern separation in the CA1 network of SC and WM animals that is not present in the IC group.

When exposed to the AA condition, WM animals present a significantly higher SiSc than both the IC and SC groups (p<0.001), indicating that in the CA1 network the most overlapping ensembles recruited by the (AA) double spatial exploration experience were found in WM animals (Fig 5A; S3 File); this suggest that a more reliable network coding was found in SC and particularly in WM-treated animals than in the IC group (Fig 5A).

We then performed a 2-way ANOVA to examine the influence of each independent variable (behavioral pre-treatment and double exploration condition before sacrifice) on the SiSc results obtained in CA1. The analysis revealed a significant effect of the pre-treatment (F _2,2_ = 14.138 p<0.001), a significant effect of the double spatial exploration (F _2,2_ = 201.33 p<0.001), and a significant interaction between these independent variables (F _4,27_ = 16.585 p<0.001). The *post hoc* analysis for the pre-treatment variable revealed that the WM animals present significant differences when compared to the IC condition (p<0.001), but the SC animals did not (p = 0.79); moreover, significant differences were found between WM and SC animals (p<0.001). The *post hoc* analysis for the double exploration condition revealed significant differences among all double exploration conditions (p values<0.001). These results showed that the most effective pre-treatment to modify CA1 network coding is the WM and confirmed that the degree of overlap between the recruited ensembles depends on the features of each double spatial exploration condition; the significant interaction suggested that pre-treatment history determines how the CA1 network reacts to the different double spatial exploration conditions.

In the CA3 network the SiSc values also differed among the different groups (F _8, 27_ = 138.26 p<0.01). After the AB condition the SiSc values obtained in the SC and WM-treated animals were significantly lower (p<0.001 and p<0.01, respectively) than those obtained in the IC group, indicating a lower degree of overlap, which can be interpret as an improvement in spatial pattern separation in the CA3 network of both WM and SC animals (Fig 5B; S4 File).

Moreover, the CA3 network from the SC and WM-pre-treated animals exposed to the AA’ exploration showed a significantly higher SiSc compared to that obtained in the same conditions in the IC group (p<0.01 and p<0.001 respectively). The SiSc values obtained in the AA and AA’ exploration from SC and WM-pre-treatment animals were similar, indicating that unlike CA1, the CA3 network recruits highly overlapping ensembles in response to slightly different input patterns and that upon a partial input, the CA3 of SC and WM-treated animals performs pattern completion.

When the animals were exposed to the same double spatial exploration condition (AA), the most overlapping ensembles were recruited in the WM-treated animals (p<0.001 vs. IC and p<0.05 vs. SC), possibly indicating that CA3 network coding is also more reliable after WM pre-treatment (Fig 5B).

The 2-way ANOVA performed on the SiSc results obtained in CA3 revealed a significant effect of the pre-treatment condition (F _2,2_ = 11.60 p<0.001), a significant effect of the double exploration condition (F _2,2_ = 237.56 p<0.001), and a significant interaction between them (F _4,27_ = 18.98 p<0.001). The *post hoc* analysis for the pre-treatment effect showed that the WM animals presented significant differences when compared to the IC group (p<0.001), but the SC and IC animals did not differ significantly (p = 0.063). Also, significant differences were found between WM and SC animals (p<0.05). The *post hoc* analysis for the double exploration condition revealed significant differences among all double exploration conditions (AA vs. AA’ p<0.05; all other p values<0.001). This indicated that WM treatment significantly improves network coding reliability (higher overlap after AA), spatial pattern completion (higher overlap after AA’), and pattern separation (lower overlap after AB) in the CA3 network.

Although the 2-way ANOVA for the SiSc measure in both the CA1 and CA3 networks revealed that the main effect is observed in the WM-treated animals, the interaction indicates that both the SC and WM pre-treatments affect the way the network responds to the double exploration.

### Synaptophysin/Map2 staining analysis

We had previously observed, using Timm staining, that WM training in rats promotes MF expansion in to their CA3 stratum oriens [2, 3]. This was confirmed with electron-microscopy [2], and later on with more elegant and powerful imaging tools [6, 7]. This has also been demonstrated with the immunostaining for synaptophysin [4], and since an immunostaining for synaptophysin is compatible with our fresh frozen tissue obtained for catFISH, we decided to evaluate the possible MF expansion using this histological approach, in order to do it in the same tissue from the animals used for catFISH.

Then, in order to confirm that the WM-training experience induces structural synaptic plasticity, such as MF expansion, in the hippocampus [2, 3, 4, 5], adjacent brain sections from the animals used for the catFISH analysis were double stained for synaptophysin and Map2. Synaptophysin is a protein present in synaptic vesicles, and its immunostaining reveals pre-synaptic boutons in both granular cells and pyramidal neurons [4, 28, 29], while the MAP2 staining was used to identify pyramidal cell dendrites [30, 31].

For this analysis the animals were separated into the 3 pre-treatment groups (IC n = 16; SC n = 13; and WM n = 13), each of which includes animals exposed to the 3 different spatial-exploration conditions and at least one cage control used in the catFISH analysis.

**Fig 8:**
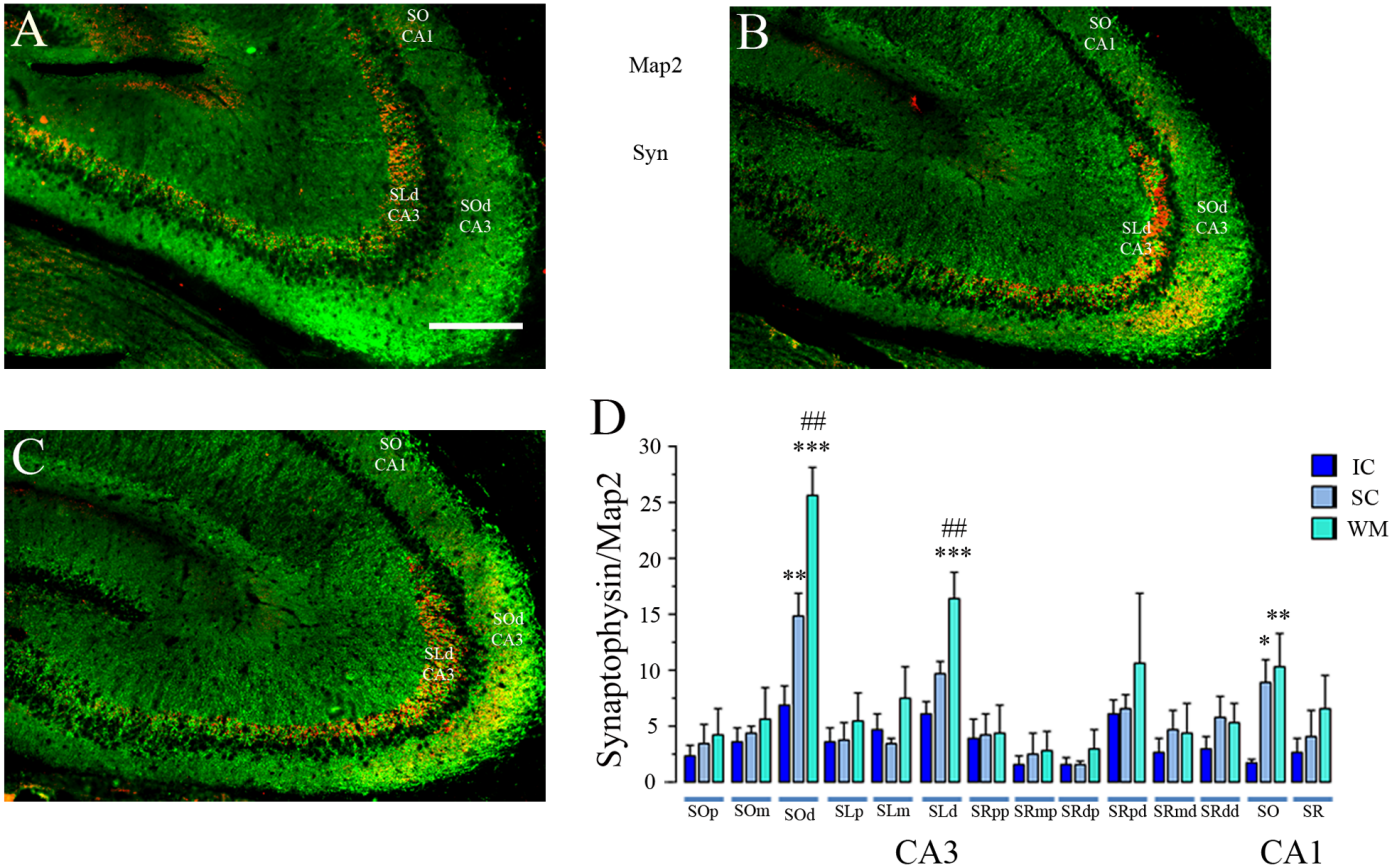
Synaptophysin-staining analysis in the hippocampus. Representative MosaiX montage images that covers all the CA3 and part (after trimming) of the CA1 hippocampal regions: in green is the Map-2 immunostaining and in red the synaptophysin staining in animals from the IC (A), SC (B), and WM (C) groups; the white bar represents 500 μm. (D) The bars represent the average synaptophysin/Map2 staining (±SEM) in each group (IC, SC, and WM) for each ROI (see Methods). Note that only in the CA3 *SOd* (*stratum oriens distal*), the CA3 *SLd* (*stratum lucidum distal*), and the CA1 *SO* (*stratum oriens*) were significant differences found between groups. Fisher *post hoc* analysis of the WM vs. the IC ***p<0.001, **p<0.01, *p<0.05; WM vs. the SC ##p<0.01.

The analysis of the synaptophysin-stained area in the different ROIs revealed statistically significant differences between groups only in 3 regions (Fig 8; S5 File): the CA3 *stratum oriens* distal (*SOd*) relative to the DG (F _2, 39_ = 24.32 p<0.001), the CA3 *stratum lucidum* distal (*SLd*) relative to the DG (F _2, 39_ = 10.77 p<0.001), and in the CA1 *stratum oriens* (*SO*: F _2, 39_ = 6.05 p<0.01). In the CA3-*SOd*, the animals from the WM group presented a significantly larger synaptophysin-staining area than the IC group (Fisher p<0.001); however, we also found significant differences between the SC and IC groups (p<0.01). Importantly, here we also found significant differences between the WM and SC groups (p<0.01), revealing that the WM treatment induced a more robust MF expansion. In the CA3-*SLd* we also found a significantly higher density of synaptophysin staining in WM animals than in the SC and IC groups (p<0.001), and no significant differences were found here between these latter two groups. Since the CA3-*SOd* and particularly the CA3-*SLd* are targets of MF boutons, increased synaptophysin staining here strongly suggests MF expansion [4] in the SC- but more robustly in WM-treated animals (Fig 8).

Finally, the CA1-*SO* from animals that underwent either the WM or SC pre-treatment showed a significantly larger synaptophysin-stained area compared to that of IC animals (p<0.01 and p<0.05 respectively). No differences were found between the WM and SC animals. The CA1-*SO* is a target of Schaffer collaterals, and this result suggests that both the WM and SC experiences induced a greater density of Shaffer collaterals in the CA1-*SO* (Fig 8).

### The synaptophysin-stained area in CA3 significantly correlates with measures of pattern separation and pattern completion

Since the SiSc depends on the double exploration condition used for the catFISH analysis, the correlation analysis was done between the synaptophysin-stained area and the SiSc obtained in each of the 3 different conditions (AA, AA’, and AB). This analysis included all animals from all pre-treatment groups (WM, SC, and IC).

**Fig 9:**
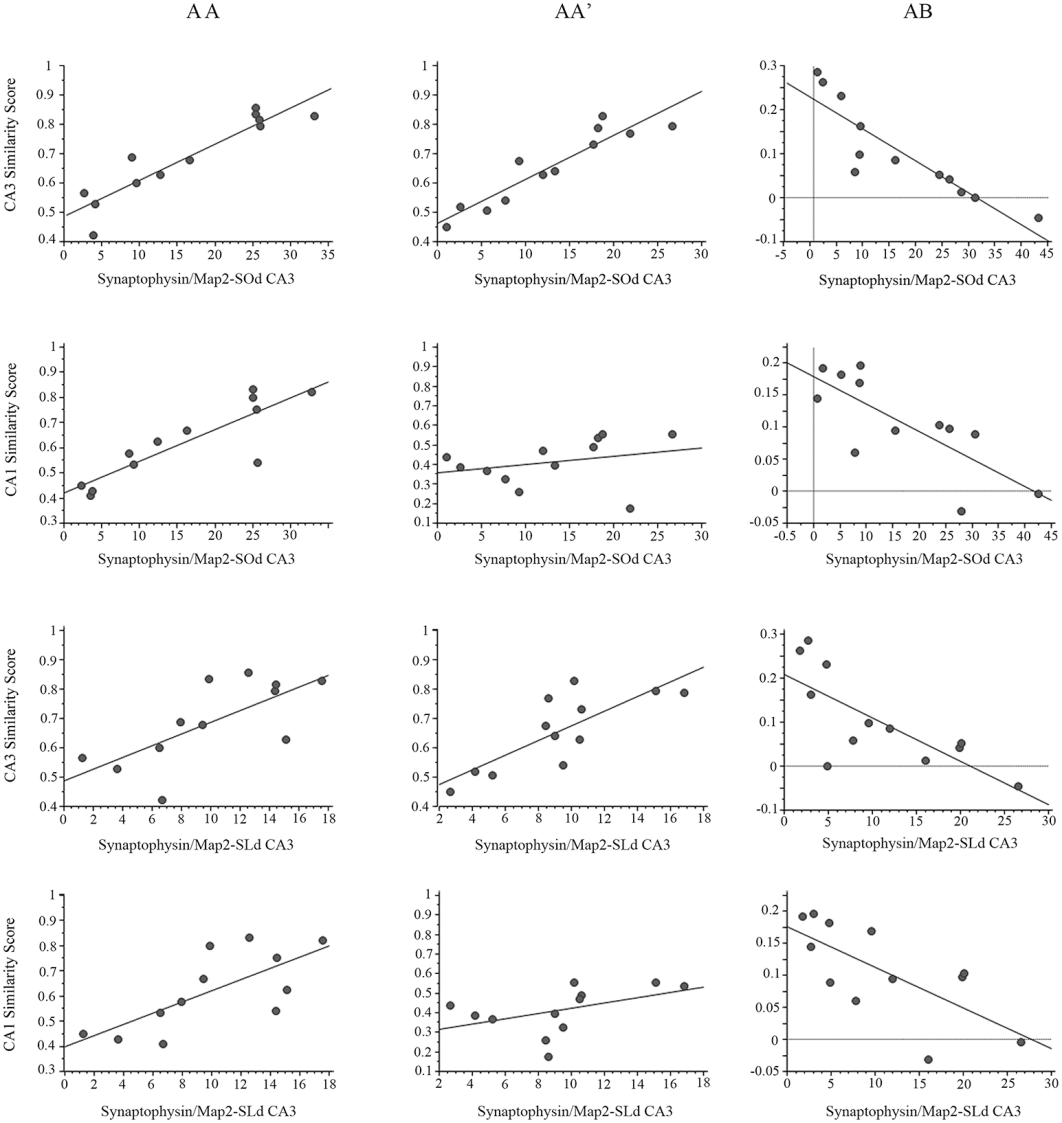
Synaptophysin/Map2 area and Similarity score correlation analysis. Correlation graphs between: the area of synaptophysin staining in the CA3 *SOd* and the similarity scores (SiSc) obtained in the CA3 network (first row); the area of synaptophysin staining in the CA3 *SOd* and the SiSc obtained in the CA1 network (second row); the area of synaptophysin staining in the CA3 *SLd* and the SiSc obtained in the CA3 network (Third row); and the area of synaptophysin staining in the CA3 *SLd* and the SiSc obtained in the CA1 network (Fourth row). The first column presents the correlation graphs for the AA double exploration condition, where pattern completion is observed in both the CA3 and CA1 networks; the second column presents the correlation graphs for the AA’ condition, where pattern completion occurred in the CA3 network, and partial pattern separation occurred in CA1; and the third column presents the graphs for the AB condition where pattern separation occurred in both CA1 and CA3. ***p<0.001, **p<0.01, *p<0.05.

The SiSc obtained in the CA3 network showed significant, positive correlations with the synaptophysin-stained area in the CA3-*SOd* (Fig 9; S6 File) after both the AA (*r* (40) = 0.907 p<0.001) and AA’ double exploration (*r* (40) = 0.870 p<0.001), indicating that what may be primarily MF in the CA3-*SOd* correlates with a higher neural ensemble overlap in CA3, which suggests a more reliable network coding in CA3 and improved proper pattern completion.

Conversely, in the AB condition, the density of synaptophysin in the CA3*-SOd* negatively correlates with the SiSc (*r* (40) = -0.884 p<0.001), indicating that a high connectivity in a target region for MFs positively correlates with a lower degree of ensemble overlap and the recruitment of more independent ensembles, suggesting that the improved spatial pattern separation in the CA3 network is related to CA3-*SOd* structural plasticity, likely to be MF expansion.

Interestingly, the SiSc obtained after the AA double exploration in the CA1 network also shows a significant, positive correlation with the synaptophysin-stained area in the CA3-*SOd* (*r* (40) = 0.870 p<0.001), indicating that more overlapping ensembles are recruited in CA1 after the AA condition, in a network with increased density of synapses in the CA3-*SOd*. This suggests that a more reliable network coding in CA1 may also be related to MF expansion that occurred in the CA3-*SOd*. Similarly, the SiSc in the CA1 network obtained after the AB double exploration negatively correlates with the area of synaptophysin staining in the CA3-*SOd* (*r* (40) = -.767 p<0.01), indicating that less overlap or more independent ensembles are recruited in CA1 after the AB condition when the density of CA3-*SOd* synapses is higher. This agrees with the idea that pattern-separation efficacy in the CA1 network is also related to what may largely be MF expansion in the CA3-*SOd*.

When comparing the synaptophysin-stained area in the CA3-*SLd* and the SiSc obtained in the CA3 network, a significant positive correlation was found in the AA (*r* (40) = 0.639 p<0.05) but barely not in the AA’ condition (*r* (40) = 0.556 p = 0.06). In the AB condition a significant negative correlation was also found between measures (*r* (40) = -0.747 p<0.01), which indicates that as the CA3-*SLd* acquires more synapses, mostly MFs, a higher overlap is detected after the AA condition, and conversely in the AB condition when synapse density is higher in the CA3-*SLd* less overlapping ensembles or more independent ensembles are recruited. This suggests that pattern separation and coding reliability improvement in CA3 may partly result from MF expansion or increase connectivity in the CA3-*SLd*.

The correlation between the synaptophysin-stained area in the distal CA3-*SLd* and the SiSc obtained in the CA1 network was significant in the AA condition (*r* (40) = 0.623 p<0.05), and was highly significant and negative in the AB condition (*r* (40) = -0.722 p<0.01). This indicates that the higher the density of synapses in the CA3-*SLd*, the higher the overlap detected after the AA condition in CA1, while after the AB condition the higher density of synapses in CA3-*SLd* predicts a lower overlap or a more independent ensemble recruitment in CA1. This result may imply a network mechanism in which the reliability of CA1 network coding and especially pattern separation in CA1 benefit from the MF expansion in the CA3-*SLd*.

Finally, the synaptophysin staining in the CA1-*SO* showed a significant negative correlation with the SiSc in the CA3 network after the AB condition (*r* (40) = -710 p<0.05). This suggest that what might likely be an increase density of Schaffer collaterals in the CA1-*SO* correlates with a lower overlapping in CA3 after the AB condition. This is an intriguing result suggesting that the improved pattern separation in CA3 may also be related to a higher density of Schaffer collaterals in the CA1-*SO*.

## Discussion

The results from the DNMP task revealed that “*spatial experience*” provided by the WM and SC treatments improve the behavioral expression of spatial pattern separation (Fig 1). The SiSc values obtained with catFISH in the CA hippocampal regions revealed that after the animal experiences two different environments (AB), these networks recruit ensembles with a low degree of overlap. This is interpreted as a neurophysiological correlate (direct or indirect, see below) of spatial pattern separation in CA1 and CA3 that occurs particularly well in animals previously exposed to the WM treatment, but also after the SC treatment, which mostly influences the CA3 network. Interestingly the catFISH results also revealed that the SC and particularly the WM treatment improved partial pattern separation in CA1, i.e., the recruitment of gradually different ensembles after two gradually different exploration experiences (AA’ vs AA). After the AA’ double exploration condition the CA3 network from WM-treated animals showed the highest degree of overlap, suggesting an improvement in proper pattern completion. Moreover, the present results also provided evidence suggesting that a more reliable coding was promoted in both the CA1 and CA3 networks from WM-treated animals, since neuronal recruitment in these networks presented a high degree of overlap after the AA condition. The image analysis of synaptophysin/Map2 staining suggested that the WM treatment was an important factor inducing structural synaptic plasticity in the hippocampus. Moreover, we found highly significant correlations between the SiSc measures and the synaptophysin-stained areas, further suggesting that structural plasticity and possibly MF expansion underlies the improvements in hippocampal information processing functions.

The DNMP task revealed that both the SC and WM-treated animals adequately discriminate even difficult separations (Sp1 and Sp2), while the IC animals struggle already in S3 File and perform very similarly to the SC and WM groups in S4 File. Using a behavioral paradigm that evaluates short-term memory for spatial location information as a function of spatial similarity between spatial locations, Gilbert, and colleagues [32] found that the performance of animals with dentate gyrus lesions improved as a function of greater spatial separation between the objects. A similar effect was observed by Cleland [21] in mice with reduced neurogenesis that showed deficits in the DNMP task in the closer separations (Sp2, Sp3), but not in the large separation (Sp4); dentate gyrus lesions in rats produced a similar effect [33].

Gilbert and colleagues [32] concluded that the DG function is related to the efficacy of spatial pattern separation, while Morris and colleagues [33] further suggested that the role of the hippocampus, and more specifically the dentate gyrus, in spatial pattern separation is to overcome spatial interference in order to discriminate between spatial locations that share a large amount of overlap, and the system overcomes such spatial interference giving rise to pattern separation.

In a context where the issue is: How are pattern completion and pattern separation to be evaluated? it has been noted that if one can generate a clear gradient in the behavioral output that scales with the degree of “interference of the DG system, in this case”, then the differences found in the behavioural performance can be more easily inferred based on the overall pattern of deficits [34]. In the present experiments we can consider the interference of the DG system a function of the amount of previous “spatial experience” (IC vs WM, or IC vs SC, SC vs WM) which in turn, is related to incremental differences of hippocampal distal CA3 network connectivity (Figs 1, 5 and 8). It is important to note that the contextual experience in the WM condition is more complex or rich, follow by that obtained by SC animals and it is clearly less in the IC animals. Performance in the DNMP task shows gradual differences between the WM, SC and IC groups; the WM animals are the best performers and the IC are the worst, with the SC in an intermediate but clearly improved range. It is important to note that the SC animals experienced primarily swimming exercise and more subtly contextual learning.

The catFISH SiSc measure reveals a strikingly similar gradient between the WM, SC and IC treatments: less overlap or more independent ensembles after the AB double exploration, particularly in the WM pre-treated animals, in both the CA3 and CA1 networks; the SC group also present less overlap than the IC group, but only in CA1. Low overlap or ensemble independence after the AB condition in both networks indicates place cell remapping and can be interpreted as pattern separation [22, 23].

It is important to acknowledge that the catFISH method may not provide a direct read out of spatial pattern separation since, as stated by Hunsaker and Kesner [18], it is possible that the SiSc data may represent a different measure altogether of some higher-level mnemonic process that relies on pattern separation function [35, 36], although it is not clear what such a mnemonic process might actually be. Nevertheless, we should clarify that the SiSc measures that we observed after the different experiences (AB, AA, AA’) may either be a direct or indirect readout of pattern separation, and other information processing functions such as proper pattern completion, partial pattern separation and coding reliability and/or pattern completion.

Importantly, this neurophysiogical readout of pattern separation (SiSc after AB) presented an important correlation with the anatomical measure of synapse density we used here (Fig 9). This strongly suggests that increase synapse density in the distal CA3 may contribute to pattern separation efficacy in CA3, supporting the idea that the DG is related to pattern separation efficacy and suggesting that greater connectivity in distal CA3 helps to improve the spatial pattern separation function.

The DNMP task revealed that the IC animals perform the worst spatial pattern separation, and it is important to note that a high number of errors in the DNMP task in Cleland’s study [21] was previously interpreted as impaired spatial pattern separation. The SiSc results obtained by catFISH further support this idea. After the AB double exploration, IC animals present the highest degree of overlap, indicating poor spatial pattern separation. The SiSc results obtained after AA’ revealed that in both WM and SC animals, CA1 ensemble recruitment changes in response to partial variations in the environment, that is, CA1 reacts to altered input patterns by recruiting gradually different ensembles, creating an altered output representation which can be considered as partial remapping [26] or partial pattern separation. Note that this does not occur in IC animals, whose CA1 network fails to show a clear partial pattern separation upon partial variations in the input (AA’). Meanwhile, the CA3 of IC animals show the lowest degree of overlap after the AA’ condition, suggesting poor proper-pattern completion.

Along this line of thinking, our catFISH results obtained in both WM and SC animals are more similar to those obtained in previous reports using either electrophysiology or catFISH [23, 25, 37] in animals that had been previously trained or habituated to spatial exploration. In contrast, the SiSc results obtained in our IC animals resemble those obtained in impaired conditions [38]. This hippocampal dysfunction in IC animals may result from their isolation [39], which is known to produce deficits in hippocampus-dependent tasks and to decrease hippocampal BDNF expression [40], an important factor for circuit rewiring [41]. Computational modeling showed that the sparse MF connectivity benefits CA3 function, but a vanishingly sparse connectivity may lead to network dysfunction [12].

The hippocampal network architecture provides a competitive neural network in the dentate gyrus that precedes the CA3 auto-association system. This is a critical feature, since without the orthogonalization provided via pattern separation by the DG, the CA3 auto-associative network is presented with a mixture of inputs (instead of separated) that could produce a mixed output state which would lead to “catastrophic interference” [18]. The network would therefore be incapable of storing separate memories due to insurmountable levels of interference among stimuli, and subjects would fail at the learning task.

During development, the MF projections into the CA3-*SO* are abundant, but in individually caged Wistar Rats the MFs retract approximately 24 days after birth, and both retraction and growth are input dependent [28]. When rats that had experienced a spatial learning task during puberty, MF expansion into the CA3-*SOd* was detected in the adult, and these adult animals showed that further spatial learning was improved [42]. Different rat strains and closely related vole species occupying different habitats present different densities of MFs in the CA3-*SO*, which correlate with their spatial abilities [43, 44]. These features of the MF projection suggest that poor environmental conditions may lead to MF atrophy and consequently, to poor hippocampal information processing and behavioral impairment.

These features of the MF projection suggest that poor environmental conditions may lead to MF atrophy and consequently, to poor hippocampal information processing and behavioral impairment. Remarkably, our results suggest that in the adult rat this impairment may be reversed by proper hippocampal stimulation that improves hippocampal connectivity and information processing. This idea may well lead to suitable treatments for various cognitive pathologies [18, 19], as well as for neurocognitive impairments in aging [20]. Here and elsewhere [38] we have found that pattern completion improvement is accompanied by good pattern separation. One possible explanation is that the anatomical remodeling, detected here in the CA3 and CA1 regions, may simultaneously impact several hippocampal information-processing mechanisms, as suggested by our SiSc measures. It is also possible that a good pattern separation during encoding facilitates further decoding during retrieval, i.e., “a high efficacy of pattern separation enhances encoding and improves the ability of temporarily remembering one spatial location as separate from another” [18].

We need to clarify the meaning of the SiSc measures obtained after the AA double exploration condition (same room, same box). One possible interpretation is that it measures “coding reliability”. After a double spatial exploration in the same room and box conditions (AA) the animals recruit highly overlapping ensembles in both the CA3 and CA1 networks, particularly those pre-treated as WM, which are likely to have a high synapse density in the distal CA3 dendrites. Congruently, less overlap is seen in the IC, which presented the lowest synapse density. Importantly, this ensemble overlap after the AA condition correlates with the synaptophysin staining area in the distal CA3 region (Fig 9]. This suggests that the anatomical conditions that appear to improve spatial pattern separation in the hippocampal network (as discussed above) also improved spatial pattern completion. We also found that the network is recruiting, with high fidelity, the same neurons twice after having experienced the same environment twice (AA). The SiSc measures the degree of overlap or similarity between the ensembles recruited by the first and second exploration, and trial-to-trial neuronal activity variability in response to repeated presentation of the same stimulus is often quantified in terms of reliability [45]. If the same number of spikes is emitted from one trial to the next, the neuron is said to be reliable [46]. Similarly, in hippocampal neurons a strong pattern of activity must be reinstated in order to drive *Arc* mRNA expression for a second time in the CA1 and CA3 networks [24, 47]. The SiSc measure obtained after the AA condition provides some information on the reliability of both encoding and decoding. The network achieves higher coding reliability because its neurons communicate shared information with each other, whereas the independent neurons do not [48], suggesting that the reliability may reflect the strength or efficiency of ensemble formation. Nevertheless this interpretation needs to be supported by conclusive evidence revealing the patterns of neural activity that drives Arc expression over and over again, which is not clear yet. For this reason, it is important to consider that the mechanism through which the hippocampus facilitates memory retrieval is pattern completion [49, 50]. However, the current definition of pattern completion states that this mechanism is triggered when the animal is exposed to a partial or degraded retrieval cue [18]. This is what previous studies had shown to occur in the CA3 hippocampal network, which recruits highly overlapping ensembles after the animal experiences minor variations in the environment [16, 23, 25], and this was interpreted here as proper pattern completion. It is an important advantage for a memory system to be able to recall a previously stored pattern upon a partial or degraded input, but in real life, animals are exposed to stimuli presented in a graded manner. As more similarities are detected between the new input and the already stored representation, the more likely it is that the system will retrieve the previously stored pattern. With this rationale, it is possible that the system evaluation of the input patterns may bring a result that falls below the similarity threshold for ensemble recruitment; but in other situations, the threshold may be surpassed far beyond its limit, with an input pattern of 90% or more similarity, promoting pattern completion. In the AA condition the same pattern completion mechanism must be triggered, possibly with a higher fidelity.

The synaptophysin-staining results revealed that structural synaptic plasticity occurs as a consequence of *“spatial experience”*, particularly after WM training. The increased density of synaptophysin in the CA3-*SOd* may reveal the increased connectivity of several inputs, such as septal fibers, commissural fibers from the contralateral hippocampus, and particularly CA3 recurrent connections which target the CA3-*SOd* region. However, the size of MFs and the fact that it has been previously demonstrated that MF expand in this region [2, 3, 4, 5] suggest that the increase in synaptophysin staining likely represents MF expansion. Moreover, the increased density of synaptophysin observed in the CA3-*SLd* of WM-treated animals can be considered more compelling evidence of MF expansion [6]. In this regard, increase density of synaptophysin in the CA3-*SOd* was also evident in the SC animals. This contrasts with previous findings [2, 3], but in the present experiment we used a different behavioral room with different conditions (i.e., lighting) that may have promoted uncontrolled contextual learning [51]. In both treatments (WM and SC) the animals underwent a spatial behavioral experience that included exercise (swimming) and context representation, and it has been observed that extended exercise (see Methods) improves hippocampal function through a BDNF-dependent mechanism [52, 53] and induces MF expansion [54]. Swimming exercise alone (without a memory task) can stimulate CA3 pyramidal cell activity patterns [55] and this in turn may induce the MF structural plasticity observed here in the SC animals, as reported previously [56, 57]. This may suggest that swimming exercise itself may be able to improve spatial pattern recognition. Note however that the MF expansion was significantly smaller in SC than in the WM-overtrained animals; this strongly suggests that a cognitive component that is more salient or present exclusively in the WM condition, such as motivated spatial learning, may be required to promote a robust structural plasticity in the CA3 hippocampal region.

A significant increase in the synaptophysin-stained area was also found in the CA1-*SO* region of WM and SC animals. Similarly, increased density of spines in the CA1-*SO* was observed after rats experienced a complex environment [58] or exercise [59]. These results suggest that both MF expansion and the increased density of Schaffer collaterals are induced by *“spatial experience”*.

It is important to acknowledge the fact that many other plastic changes may occur in the rat’s brain; however, the highly significant correlation between the SiScs obtained in both CA networks and the synaptophysin area in the CA3-*SOd* suggest that is likely that the MF projections may play a prominent role in spatial pattern recognition and suggests that optimization of the CA3 network modulates CA1 function [60].

Our evidence points to a possible role for MFs not only in pattern separation but also in pattern completion in CA3. The CA3 network operates as an auto-associative memory system storing spatial information. This network receives information from the entorhinal cortex (EC-L2) through the perforant path (PP) projection, which provides ~4000 synapses/neuron, and indirectly through the DG network, which receives the same *en passant* projections from the PP but provides a sparse input, with only ~50 synapses/neuron to the CA3 [10, 61]. Thus, duplicate information is conveyed from the EC to the CA3 [13]. Moreover, each CA3 pyramidal cell receives ~12,000 recurrent collateral synapses [61]. Based on two prominent features, their size and their target location on the pyramidal cell dendrite, MFs can be considered reliable triggers, or “detonators”, of pyramidal CA3 cell activity [62]. This notion has led computational models to suggest a role of MFs in promoting spatial pattern separation to encode information in CA3 [13] and is supported by evidence showing that inactivation of the MF pathway blocks encoding, but not retrieval [14]. Abundant evidence supports the relevance of the MFs in spatial and non-spatial hippocampal pattern separation [15, 16].

The role of MFs in pattern completion was previously proposed theoretically [10, 63], and the present results may represent experimental evidence supporting it. Other features of the MF projection, in addition to those considered in defining them as “detonators”, can help to understand its role in pattern completion. For example, the number of MF synapses reaching the dendritic shaft of GABAergic interneurons in the CA3-*SL* greatly exceeds the number of MF synapses reaching pyramidal neurons [64]; the MFs have multiple release sites, and they carry several neurotransmitters and neuromodulators that regulate NMDA receptor function [65]. Therefore, when computing the average synaptic currents of each of the 3 inputs reaching CA3 pyramidal cells, the recurrent collaterals can be those providing the most information [63], but this condition varies across the different input areas and depends on the activity state of the network. This is compatible with the idea that the MFs are not only a reliable transmitter device, but may also be information-processing units [10], switching between a minimal-impact "off" phase when inhibition dominates and an “on” phase when MFs reliably trigger spikes in CA3 pyramidal neurons. Thus, the MF can be considered “a conditional detonator” or “a discriminator” [63]. This way, upon experiencing a set of stimuli, either fully or partially associated with a familiar episode, our CA3 neuronal ensemble storing this information will be recruited by the PP synapses when the MF input is switched “off”, allowing the CA3-CA3-potentiated synapses to prevail and complete the original pattern. Likewise, inhibiting the output of “old granule cells” impairs spatial pattern completion [66]. Additionally, pattern completion requires synaptic plasticity in the CA3-CA3 network [49, 67], and burst stimulation of the MF induces heterosynaptic LTP in CA3-CA3 synapses [68]. Pattern separation, on the other hand, is achieved through a “winner-take-all" mechanism [10] during the “on” phase, when MFs are “detonators”, driving information storage in CA3 [12].

Finally, MF expansion on terminals reaching CA3 interneurons improves memory precision [7]; this can perhaps be explained by our evidence suggesting that hippocampal information processing depends on an optimal MF projection.

## Supporting Information

S1 File Water Maze data set. (SVD)

S2 File Delay Non-Matching to Place Data Set. (SVD)

S3 File Compartamental analysis of temporal activity using FISH forArc in the CA1 region. (SVD)

S4 File Compartamental analysis of temporal activity using FISH forArc in the CA1 region. (SVD)

S5 File Map2andSynaptophysing staining analysis. (SVD)

S6 File Map2andSynaptophysingvs CA1 and CA3 similarity score correlation analysis. (SVD)

## References

[1] Caroni P, Donato F, Muller D. Structural plasticity upon learning: regulation and functions. Nat Rev Neurosci. 2012; 13: 478–90. 10.1038/nrn3258 22714019

[2] Ramírez-Amaya V, Escobar ML, Chao V, Bermúdez-Rattoni F. Synaptogenesis of mossy fibers induced by spatial water maze overtraining. Hippocampus. 1999; 9: 631–6. 10641755 10.1002/(SICI)1098-1063(1999)9:6<631::AID-HIPO3>3.0.CO;2-3

[3] Ramírez-Amaya V, Balderas I, Sandoval J, Escobar ML, Bermúdez-Rattoni F. Spatial long-term memory is related to mossy fiber synaptogenesis. J Neurosci. 2001; 21: 7340–8. 11549744 10.1523/JNEUROSCI.21-18-07340.2001 PMC6763009

[4] Holahan MR, Rekart JL, Sandoval J, Routtenberg A. Spatial learning induces presynaptic structural remodeling in the hippocampal mossy fiber system of two rat strains. Hippocampus. 2006;16: 560–70. 16685708 10.1002/hipo.20185

[5] Middei S, Vetere G, Sgobio C, Ammassari-Teule M. Landmark-based but not vestibular-based orientation elicits mossy fiber synaptogenesis in the mouse hippocampus. Neurobiol Learn Mem. 2007; 87: 174–80. 16990035 10.1016/j.nlm.2006.08.004

[6] Galimberti I, Gogolla N, Alberi S, Santos AF, Muller D, Caroni P. Long-term rearrangements of hippocampal mossy fiber terminal connectivity in the adult regulated by experience. Neuron. 2006; 50: 749–63. 16731513 10.1016/j.neuron.2006.04.026

[7] Ruediger S, Vittori C, Bednarek E, Genoud C, Strata P, Sacchetti B, Caroni P. Learning-related feedforward inhibitory connectivity growth required for memory precision. Nature. 2011; 473: 514–8. 10.1038/nature09946 21532590

[8] Hebb DO. The Organization of Behavior: A Neuropsychological Theory. John Wiley & Sons New York, USA press. 1945.

[9] Bailey CH, Kandel ER. Synaptic remodeling, synaptic growth and the storage of long-term memory in Aplysia. Prog Brain Res. 2008; 169: 179–98. 10.1016/S0079-6123(07)00010-6 18394474

[10] Bischofberger J, Engel D, Frotscher M, Jonas P. Timing and efficacy of transmitter release at mossy fiber synapses in the hippocampal network. Pflugers Arch. 2006; 453: 361–72. 16802161 10.1007/s00424-006-0093-2

[11] Chawla MK, Guzowski JF, Ramirez-Amaya V, Lipa P, Hoffman KL, Marriott LK, Worley PF, McNaughton BL, Barnes CA. Sparse, environmentally selective expression of Arc RNA in the upper blade of the rodent fascia dentata by brief spatial experience. Hippocampus. 2005; 15: 579–86. 15920719 10.1002/hipo.20091

[12] Cerasti E, Treves A. How informative are spatial CA3 representations established by the dentate gyrus? PLoS Comput Biol. 2010; 6: e1000759 10.1371/journal.pcbi.1000759 20454678 PMC2861628

[13] Treves A, Rolls ET. Computational constraints suggest the need for two distinct input systems to the hippocampal CA3 network. Hippocampus. 1992; 2:189–99. 1308182 10.1002/hipo.450020209

[14] Lassalle JM, Bataille T, Halley H. Reversible inactivation of the hippocampal mossy fiber synapses in mice impairs spatial learning, but neither consolidation nor memory retrieval, in the Morris navigation task. Neurobiol Learn Mem. 2000; 73: 243–57. 10775494 10.1006/nlme.1999.3931

[15] Lee I, Kesner RP. Encoding versus retrieval of spatial memory: double dissociation between the dentate gyrus and the perforant path inputs into CA3 in the dorsal hippocampus. Hippocampus. 2004; 14: 66–76. 15058484 10.1002/hipo.10167

[16] Kesner RP. An analysis of the dentate gyrus function. Behav Brain Res. 2013; 254:1–7. 10.1016/j.bbr.2013.01.012 23348108

[17] Yassa MA, Stark CE. Pattern separation in the hippocampus. Trends Neurosci. 2011; 34: 515–25. 10.1016/j.tins.2011.06.006 21788086 PMC3183227

[18] Hunsaker MR, Kesner RP. The operation of pattern separation and pattern completion processes associated with different attributes or domains of memory. Neurosci Biobehav Rev. 2013; 37: 36–58. 10.1016/j.neubiorev.2012.09.014 23043857

[19] Hanson JE, Madison DV. Imbalanced pattern completion vs. separation in cognitive disease: network simulations of synaptic pathologies predict a personalized therapeutics strategy. BMC Neurosci. 2010; 11, 96 10.1186/1471-2202-11-96 20704756 PMC2931521

[20] Burke SN, Wallace JL, Nematollahi S, Uprety AR, Barnes CA. Pattern separation deficits may contribute to age-associated recognition impairments. Behav Neurosci. 2010; 124: 559–73. 10.1037/a0020893 20939657 PMC3071152

[21] Clelland CD, Choi M, Romberg C, Clemenson GD Jr, Fragniere A, Tyers P, et al A functional role for adult hippocampal neurogenesis in spatial pattern separation. Science. 2009; 325: 210–3. 10.1126/science.1173215 19590004 PMC2997634

[22] Guzowski JF, Knierim JJ, Moser EI. Ensemble dynamics of hippocampal regions CA3 and CA1. Neuron. 2004; 44: 581–4. 15541306 10.1016/j.neuron.2004.11.003

[23] Vazdarjanova A, Guzowski JF. Differences in hippocampal neuronal population responses to modifications of an environmental context: evidence for distinct, yet complementary, functions of CA3 and CA1 ensembles. J Neurosci. 2004; 24, 6489–96. 15269259 10.1523/JNEUROSCI.0350-04.2004 PMC6729865

[24] Guzowski JF, McNaughton BL, Barnes CA, Worley PF. Environment-specific expression of the immediate-early gene Arc in hippocampal neuronal ensembles. Nat Neurosci. 1999; 2: 1120–4. 10570490 10.1038/16046

[25] Leutgeb S, Leutgeb JK, Treves A, Moser MB, Moser EI. Distinct ensemble codes in hippocampal areas CA3 and CA1. Science. 2004; 305:1295–8. 15272123 10.1126/science.1100265

[26] Hayman RM, Jeffery KJ. How heterogeneous place cell responding arises from homogeneous grids—a contextual gating hypothesis. Hippocampus. 2008; 18: 1301–13. 10.1002/hipo.20513 19021264

[27] Vazdarjanova A, Ramirez-Amaya V, Insel N, Plummer TK, Rosi S, Chowdhury S, et al Spatial exploration induces ARC, a plasticity-related immediate-early gene, only in calcium/calmodulin-dependent protein kinase II-positive principal excitatory and inhibitory neurons of the rat forebrain. J Comp Neurol. 2006; 498:317–29. 16871537 10.1002/cne.21003

[28] Holahan MR, Honegger KS, Routtenberg A. Expansion and retraction of hippocampal mossy fibers during postweaning development: strain-specific effects of NMDA receptor blockade. Hippocampus. 2007; 17: 58–67. 17143904 10.1002/hipo.20242

[29] Pozzo-Miller LD, Inoue T, Murphy DD. Estradiol increases spine density and NMDA-dependent Ca2+ transients in spines of CA1 pyramidal neurons from hippocampal slices. J Neurophysiol. 1999; 81: 1404–11. 10085365 10.1152/jn.1999.81.3.1404

[30] Cáceres A, Banker G, Steward O, Binder L, Payne M. MAP2 is localized to the dendrites of hippocampal neurons which develop in culture. Brain Res. 1984; 315: 314–8. 6722593 10.1016/0165-3806(84)90167-6

[31] Di Stefano G, Casoli T, Fattoretti P, Balietti M, Grossi Y, Giorgetti B, et al Level and distribution of microtubule-associated protein-2 (MAP2) as an index of dendritic structural dynamics. Rejuvenation Res. 2006; 9: 94–8. 16608403 10.1089/rej.2006.9.94

[32] Gilbert P.E., Kesner R.P., Lee I. Dissociating hippocampal subregions: double dissociation between dentate gyrus and CA1. Hippocampus. 2001; 11: 626–636. 11811656 10.1002/hipo.1077

[33] Morris A.M., Churchwell J.C., Kesner R.P., Gilbert P.E. Selective lesions of the dentate gyrus produce disruptions in place learning for adjacent spatial locations. Neurobiology of Learning and Memory. 2012; 97: 326–331. 10.1016/j.nlm.2012.02.005 22390856 PMC4089983

[34] Lacy J.W., Yassa M.A., Stark S.M., Muftuler L.T., Stark C.E. Distinct pattern separation related transfer functions in human CA3/dentate and CA1 revealed using high-resolution fMRI and variable mnemonic similarity. Learning and Memory. 2011; 18: 15–18. 10.1101/lm.1971111 21164173 PMC3023966

[35] Aimone J.B., Deng W., Gage F.H. Resolving new memories: A critical look at the dentate gyrus, adult neurogenesis, and pattern separation. Neuron. 2011;70: 589–596. 10.1016/j.neuron.2011.05.010 21609818 PMC3240575

[36] Yassa M.A., Stark C.E. Pattern separation in the hippocampus. Trends in Neurosciences. 2011; 34: 515–525. 10.1016/j.tins.2011.06.006 21788086 PMC3183227

[37] Lee I, Yoganarasimha D, Rao G, Knierim JJ. Comparison of population coherence of place cells in hippocampal subfields CA1 and CA3. Nature. 2004; 430:456–9. 15229614 10.1038/nature02739

[38] Rosi S, Ramirez-Amaya V, Vazdarjanova A, Esparza EE, Larkin PB, Fike JR, Wenk GL, Barnes CA. Accuracy of hippocampal network activity is disrupted by neuroinflammation: rescue by memantine. Brain. 2009; 132: 2464–77. 10.1093/brain/awp148 19531533 PMC2732266

[39] Fone KC, Porkess MV. Behavioural and neurochemical effects of post-weaning social isolation in rodents-relevance to developmental neuropsychiatric disorders. Neurosci Biobehav Rev. 2008; 32: 1087–102. 10.1016/j.neubiorev.2008.03.003 18423591

[40] Han X, Wang W, Xue X, Shao F, Li N. Brief social isolation in early adolescence affects reversal learning and forebrain BDNF expression in adult rats. Brain Res Bull. 2011; 86: 173–8. 10.1016/j.brainresbull.2011.07.008 21801814

[41] Park H, Poo MM. Neurotrophin regulation of neural circuit development and function. Nat Rev Neurosci. 2013; 14: 7–23. 10.1038/nrn3379 23254191

[42] Keeley RJ, Wartman BC, Häusler AN, Holahan MR. Effect of juvenile pretraining on adolescent structural hippocampal attributes as a substrate for enhanced spatial performance. Learn Mem. 2010; 17: 344–54. 10.1101/lm.1849910 20592053

[43] Schwegler H, Mueller GG, Crusio WE, Szemes L, Seress L. Hippocampal morphology and spatially related behavior in Long-Evans and CFY rats. Hippocampus. 1993; 3: 1–7. 10.1002/hipo.450030102 8364679

[44] Pleskacheva MG, Wolfer DP, Kupriyanova IF, Nikolenko DL, Scheffrahn H, Dell'Omo G, Lipp HP. Hippocampal mossy fibers and swimming navigation learning in two vole species occupying different habitats. Hippocampus. 2000; 10: 17–30. 10706213 10.1002/(SICI)1098-1063(2000)10:1<17::AID-HIPO2>3.0.CO;2-O

[45] Masquelier T. Neural variability, or lack thereof Front. Comput. Neurosci. 2013; 7:7 10.3389/fncom.2013.00007 PMC3580760 23444270

[46] Tiesinga P., Fellous J.M.,and Sejnowski T.J. Regulation of spike timing in visual cortical circuits. Nat. Rev. Neurosci. 2008; 9: 97–107. 10.1038/nrn2315 18200026 PMC2868969

[47] Kawashima T, Okuno H, Nonaka M, Adachi-Morishima A, Kyo N, Okamura M, Takemoto-Kimura S, Worley PF, Bito H. Synaptic activity-responsive element in the Arc/Arg3.1 promoter essential for synapse-to-nucleus signaling in activated neurons. Proc Natl Acad Sci U S A. 2009; 106:316–21. 10.1073/pnas.0806518106 19116276 PMC2629236

[48] Boerlin M, Machens CK, Denève S. Predictive coding of dynamical variables in balanced spiking networks. PLoS Comput Biol. 2013; 9: e1003258 10.1371/journal.pcbi.1003258 24244113 PMC3828152

[49] Marr D. Simple memory: a theory for archicortex. Philos Trans R Soc Lond B Biol Sci. 1971; 262: 23–81. 4399412 10.1098/rstb.1971.0078

[50] Willshaw D.J., Buckingham J.T. An assessment of Marr’s theory of the hippocampus as a temporary memory store. Philosophical Transactions of the Royal Society of London. Series B: Biological Sciences. 1990; 329: 205–215. 10.1098/rstb.1990.0165 1978365

[51] Tolman EC. Cognive maps in rats and men. The Psychological Review. 1948; 55: 189–208. 18870876 10.1037/h0061626

[52] Bekinschtein P, Oomen CA, Saksida LM, Bussey TJ. Effects of environmental enrichment and voluntary exercise on neurogenesis, learning and memory, and pattern separation: BDNF as a critical variable? Semin Cell Dev Biol. 2011; 22: 536–42. 10.1016/j.semcdb.2011.07.002 21767656

[53] Khabour OF, Alzoubi KH, Alomari MA, Alzubi MA. Changes in spatial memory and BDNF expression to simultaneous dietary restriction and forced exercise. Brain Res Bull. 2013; 90: 19–24. 10.1016/j.brainresbull.2012.08.005 23000024

[54] Toscano-Silva M, Gomes da Silva S, Scorza FA, Bonvent JJ, Cavalheiro EA, Arida RM. Hippocampal mossy fiber sprouting induced by forced and voluntary physical exercise. Physiol Behav. 2010; 101: 302–8. 10.1016/j.physbeh.2010.05.012 20515703

[55] Badowska-Szalewska E, Klejbor I, Cecot T, Spodnik JH, Moryś J. Changes in NGF/c-Fos double staining in the structures of the limbic system in juvenile and aged rats exposed to forced swim test. Acta Neurobiol Exp (Wars). 2009; 69: 448–58. 20048762 10.55782/ane-2009-1756

[56] Toscano-Silva M, Gomes da Silva S, Scorza FA, Bonvent JJ, Cavalheiro EA, Arida RM. Hippocampal mossy fiber sprouting induced by forced and voluntary physical exercise. Physiol Behav. 2010; 101:302–8. 10.1016/j.physbeh.2010.05.012 20515703

[57] Ni H, Li C, Tao LY, Cen JN. Physical exercise improves learning by modulating hippocampal mossy fiber sprouting and related gene expression in a developmental rat model of penicillin-induced recurrent epilepticus. Toxicol Lett. 2009; 191:26–32. 10.1016/j.toxlet.2009.07.028 19666089

[58] Moser MB, Trommald M, Andersen P. An increase in dendritic spine density on hippocampal pyramidal cells following spatial learning in adult rats suggests the formation of new synapses. Proc Natl Acad Sci USA. 1994; 91: 12673–12675. 7809099 10.1073/pnas.91.26.12673 PMC45501

[59] Stranahan AM, Khalil D, Gould E. Running induces widespread structural alterations in the hippocampus and entorhinal cortex. Hippocampus. 2007; 17: 1017–22. 17636549 10.1002/hipo.20348 PMC2956984

[60] Hoang LT, Kesner RP. Dorsal hippocampus, CA3, and CA1 lesions disrupt temporal sequence completion. Behav Neurosci. 2008; 122: 9–15. 10.1037/0735-7044.122.1.9 18298244

[61] Amaral DG, Ishizuka N, Claiborne B. Neurons, numbers and the hippocampal network. Prog Brain Res. 1990; 83: 1–11. 10.1016/s0079-6123(08)61237-6 2203093

[62] McNaughton BL, Morris RG. Hippocampal synaptic enhancement and information-storage within a distributed memory system. Trends Neurosci. 1987; 10: 408–415.

[63] Urban NN, Henze DA, Barrionuevo G. Revisiting the role of the hippocampal mossy fiber synapse. Hippocampus. 2001; 11: 408–17. 11530845 10.1002/hipo.1055

[64] Acsády L, Kamondi A, Sík A, Freund T, Buzsáki G. GABAergic cells are the major postsynaptic targets of mossy fibers in the rat hippocampus. J Neurosci. 1998; 18: 3386–403. 9547246 10.1523/JNEUROSCI.18-09-03386.1998 PMC6792657

[65] Jaffe DB, Gutiérrez R. Mossy fiber synaptic transmission: communication from the dentate gyrus to area CA3. Prog Brain Res. 2007; 163: 109–32. 17765714 10.1016/S0079-6123(07)63006-4

[66] Nakashiba T, Cushman JD, Pelkey KA, Renaudineau S, Buhl DL, McHugh TJ, et al Young dentate granule cells mediate pattern separation, whereas old granule cells facilitate pattern completion. Cell. 2012; 149: 188–201. 10.1016/j.cell.2012.01.046 22365813 PMC3319279

[67] Hasselmo ME, Schnell E, and Barkai E. Dynamics of learning and recall at excitatory recurrent synapses and cholinergic modulation in rat hippocampal region CA3. J Neurosci. 1995; 15: 5249–5262. 7623149 10.1523/JNEUROSCI.15-07-05249.1995 PMC6577857

[68] Kobayashi K, Poo MM. Spike train timing-dependent associative modification of hippocampal CA3 recurrent synapses by mossy fibers. Neuron. 2004; 41: 445–54. 14766182 10.1016/s0896-6273(03)00873-0

