# Correction: Hippocampal Synaptic Expansion Induced by Spatial Experience in Rats Correlates with Improved Information Processing in the Hippocampus

**DOI:** 10.1371/journal.pone.0137944

**Published:** 2015-09-08

**Authors:** Mariana Carasatorre, Adrian Ochoa-Alvarez, Giovanna Velázquez-Campos, Carlos Lozano-Flores, Víctor Ramírez-Amaya, Sofía Y. Díaz-Cintra

The authors are listed out of order. Please view the correct order, affiliations, and citation here:

Mariana Carasatorre^1^, Adrian Ochoa-Alvarez^1^, Giovanna Velásquez-Campos^2,3^, Carlos Lozano-Flores^1^, Víctor Ramírez-Amaya^3^, Sofía Díaz-Cintra^1^

1 Department of “Neurobiología del Desarrollo y Neurofisiología, Institutio de Neurobiología”, Universidad Nacional Autónoma de México, Querétaro, México, 2 Department of “Neurobiología Conductual y Cognitiva, Institutio de Neurobiología, Universidad Nacional Autónoma de México”, Querétaro, México, 3 Departament of “Microbiología, Maestría en Neurometabolismo & Maestría en Nutrición Humana, Facultidad de Ciencias Naturales, Universidad Autónoma de Querétaro, Querétaro, México

Carasatorre M, Ochoa-Alvarez A, Velázquez-Campos G, Lozano-Flores C, Ramírez-Amaya V, Díaz-Cintra SY (2015) Hippocampal Synaptic Expansion Induced by Spatial Experience in Rats Correlates with Improved Information Processing in the Hippocampus. PLoS ONE 10(8): e0132676. doi:10.1371/journal.pone.0132676
